# Modulation of Expression of PVY^NTN^ RNA-Dependent RNA Polymerase (NIb) and Heat Shock Cognate Host Protein HSC70 in Susceptible and Hypersensitive Potato Cultivars

**DOI:** 10.3390/vaccines9111254

**Published:** 2021-10-29

**Authors:** Edmund Kozieł, Przemysław Surowiecki, Agnieszka Przewodowska, Józef J. Bujarski, Katarzyna Otulak-Kozieł

**Affiliations:** 1 Department of Botany, Faculty of Biology and Biotechnology, Institute of Biology, Warsaw University of Life Sciences—SGGW, Nowoursynowska Street 159, 02-776 Warsaw, Poland; 2 Institute of Biochemistry and Biophysics, Polish Academy of Sciences, Pawińskiego 5a, 02-106 Warsaw, Poland; surowiecki@ibb.waw.pl; 3 Laboratory of Potato Gene Resources and Tissue Culture, Bonin Research Center, Plant Breeding and Acclimatization Institute—National Research Institute, 76-009 Bonin, Poland; a.przewodowska@ihar.edu.pl; 4 Department of Biological Sciences, Northern Illinois University, DeKalb, IL 60115, USA; jbujarski@niu.edu

**Keywords:** plant–virus interaction, hypersensitive response, heat shock cognate 70 protein, nuclear inclusion b protein

## Abstract

Potato virus Y (PVY) belongs to the genus Potyvirus and is considered to be one of the most harmful and important plant pathogens. Its RNA-dependent RNA polymerase (RdRp) is known as nuclear inclusion protein b (NIb). The recent findings show that the genome of PVY replicates in the cytoplasm of the plant cell by binding the virus replication complex to the membranous structures of different organelles. In some potyviruses, NIb has been found to be localized in the nucleus and associated with the endoplasmic reticulum membranes. Moreover, NIb has been shown to interact with other host proteins that are particularly involved in promoting the virus infection cycle, such as the heat shock proteins (HSPs). HSP70 is the most conserved among the five major HSP families that are known to affect the plant–pathogen interactions. Some plant viruses can induce the production of HSP70 during the development of infection. To understand the molecular mechanisms underlying the interactive response to PVY^NTN^ (necrotic tuber necrosis strain of PVY), the present study focused on *StHSC70-8* and PVY^NTN^-*NIb* gene expression via localization of HSC70 and NIb proteins during compatible (susceptible) and incompatible (hypersensitive) potato–PVY^NTN^ interactions. Our results demonstrate that NIb and HSC70 are involved in the response to PVY^NTN^ infections and probably cooperate at some stages of the virus infection cycle. Enhanced deposition of HSC70 proteins during the infection cycle was associated with the dynamic induction of PVY^NTN^-*NIb* gene expression and NIb localization during susceptible infections. In hypersensitive response (HR), a significant increase in HSC70 expression was observed up to 3 days post-inoculation (dpi) in the nucleus and chloroplasts. Thereafter, between 3 and 21 dpi, the deposition of NIb decreased, which can be attributed to a reduction in the levels of both virus accumulation and PVY^NTN^-*NIb* gene expression. Therefore, we postulate that increase in the expression of both *StHSC70-8* and PVY^NTN^-*NIb* induces the PVY infection during susceptible infections. In contrast, during HRs, HSC70 cooperates with PVY^NTN^ only at the early stages of interaction and mediates the defense response signaling pathway at the later stages of infection.

## 1. Introduction

Potato virus Y (PVY) belongs to the genus Potyvirus and has been considered to be one of the 10 most economically and scientifically important plant viruses [1,2]. PVY occurs as flexuous, rod-shaped particles with a positive-stranded RNA genome. The genome contains two open reading frames (ORFs) that code 11 functional proteins [3]. The RNA genome of the virions is encapsidated by multiple copies of the coat protein (CP) and linked at the 5′ end to the VPg protein. The viral RNA encodes a single large polyprotein that is processed to produce viral proteins like P1, HC-Pro, P3, 6K1, CI, 6K2, VPg, NIaPro, NIb, and CP [4]. An additional polyprotein (P3N-PIPO) is synthesized as a result of a frameshift mutation at the P3 cistron, which has been recently shown to be generated through a polymerase slippage mechanism [5,6,7]. During PVY replication, RNA polymerase at the 5′ encoding region leads to the production of a small subpopulation of viruses that are characterized by shorter ORFs [4,8]. A large polyprotein is processed by three virus-encoded proteases (P1, HC-Pro, and Nia-Pro) into mature multifunctional proteins [9]. The potyvirus RNA-dependent RNA polymerase (RdRp) is known as the nuclear inclusion protein b (NIb) [4]. A subset of the RdRp protein is localized to the endoplasmic reticulum (ER) membranes, where RNA synthesis takes place [10,11]. The binding of RdRp to the membranes is mediated by the 6K-VPg-Pro peptide [12]. In some potyviruses, the RdRp is localized to the nucleus [13]. NIb has been described as the "most sticky" potyvirus protein, due to its ability to interact with other host proteins and promote the viral replication cycle in the host. For instance, the zucchini yellow mosaic virus interacts with the poly (A) binding protein of the host for zucchini yellow mosaic virus (ZYMV) [14], while other viruses interact with the eukaryotic elongation factors or heat shock proteins (HSPs) [15,16].

Abiotic and biotic stress factors influence the internal homeostasis of cells in plants. As postulated by Park and Seo [17], HSPs are important molecules that are responsible for protein translocation and degradation under both stress and normal conditions. These proteins are also known as molecular chaperones and regulate the synthesis of pattern recognition receptors (PRRs) and cellular proteins to combat pathogens [18,19]. In plants and animals, three out of five major HSP families are the most recognized: HSP100, HSP90, HSP70, and smaller HSP [20,21]. In particular, HSP70 is the most conserved protein family whose expression is induced not only by heat shock but also by almost all types of plant stress factors [22,23]. Plants exhibit different resistance mechanisms to respond to biotic stress. As proposed by Haq et al. [24], signal perception, transduction, and activation of the immune system, the responses which are controlled by HSP, are stress-related responses. HSPs are also involved in providing defense against bacteria, fungi, and nematodes [24]. Interestingly, the participation of HSP70 in viral replication seems to be a more complicated phenomenon because the virus does not encode inducers specific to HSP [25]. Moreover, HSP70, with its homolog heat shock cognate 70 (HSC70), can exert both positive and negative effects on plant–virus interactions. Previous research studies on potyviruses showed a strong association between HSC70 proteins and NIb during the ongoing viral replication cycle and viral complex assembly [4,16]. However, in the case of PVY^NTN^ (necrotic tuber necrosis strain of PVY), knowledge regarding the alterations that take place in HSC70 and NIb during infection in nontransgenic resistant and susceptible potato plants is lacking.

Therefore, our present study aimed to check the expression of *StHSC70-8* (heat shock cognate 70-8) gene in *Solanum tuberosum* (potato) based on the distribution/localization of HSC70 proteins in situ during the two types of PVY^NTN^–potato interactions, that is, susceptible and hypersensitive infections. Moreover, we examined the modulation of expression of the PVY^NTN^*-NIb* gene and analyzed the spatial distribution of the NIb protein in the tissues of inoculated potato leaves. The results of the study revealed that the *StHSC70-8* gene is induced at 3–21 days postinoculation (dpi) in susceptible potatoes, while in the hypersensitive potatoes, induction is observed only up to 3 dpi time point. The distribution of both PVY-NIb and HSC70 proteins paralleled the expression patterns and depended on the interaction type.

## 2. Materials and Methods

### 2.1. Plant Material, Virus Inoculation, and DAS-ELISA Test

The NTN strain of PVY (PVY^NTN^) was inoculated into the potato plants (*S. tuberosum* L.) of two cultivars: susceptible Irys (PVY^NTN^ resistance level of 5.5 on a scale of 1–9) [26] and resistant Sekwana (resistance level of 8 on a scale of 1–9) [26,27,28], which were obtained from IHAR-PIB, Plant Breeding and Acclimatization Institute, Bonin Research Center. In the case of Sekwana, the resistance gene *Ny-1* was localized on chromosome IX, a finding similar to that revealed by Rywal [29]. The procedures followed for the cultivation and inoculation of potato seedlings (at the four-leaf stage) with PVY^NTN^ were in accordance with those described in previously published papers [26,30,31]. Sekwana variety developed a hypersensitive necrotic response that was visible at 6–7 dpi. The reaction was confirmed by the presence of *the Ny-1* gene in Sekwana, which is responsible for conferring a hypersensitive response (HR) to PVY. Irys variety showed systemic necrosis which was visible at 10–15 dpi [32,33,34]. Leaves of mock- and PVY^NTN^-inoculated plants were evaluated for the presence of the virus by the DAS-ELISA technique, as described by Kozieł et al. [35], using the primary monoclonal antibodies against PVY^NTN^ (Bioreba, Reinach, Switzerland) followed by purified antirabbit secondary antibodies conjugated with alkaline phosphatase (Bioreba, Reinach, Switzerland) [36]. Each repeated experiment was performed on a new ELISA plate. For each test, samples from 25 mock-inoculated plants (both cultivars) were combined separately, and the same protocol was carried out for 25 PVY^NTN^-inoculated plants (both cultivars). All DAS-ELISA tests were performed using the same reagents. The readings of OD_405nm_ values were taken after 60 min in duplicates at 3, 7, and 21 dpi. The mean OD_405nm_ values were statistically analyzed using one-factor analysis of variance (ANOVA), as described in a publication by Kozieł et al. [35] using the Statistica software (version 13.0; StatSoft and TIBCO Software Inc., Palo Alto, CA, USA). For a more precise estimation, the corrected mean OD_405nm_ values were computed as described in [35] and used to compare the relative levels of virus presence/concentration in plants. The cut-off point was calculated using a formula suggested by Bioreba (Switzerland) [37] and Otulak-Kozieł et al. [38].

The calculated cut-off point value was 0.135. The readings at OD_405nm_ were compared to the calculated cut-off point, and all OD_405nm_ values greater than 0.135 were considered to be positive (i.e., confirmed the presence of the virus) [38]. The significant threshold/cut-off point values obtained after performing DAS-ELISA confirmed the presence of the virus in all the inoculated potato plants.

### 2.2. Isolation of RNA and Genomic DNA (gDNA) for Expression Analysis of Plant PVY^NTN^-NIb and StHsc70-8 in PVY^NTN^-Infected Potato Plants

To localize the plant host HSP (HSC70) and the virus NIb protein, molecular analyses were performed by collecting the samples at the same time intervals as those used for microscopic localization studies. Leaf samples (0.1 g of each sample) were collected from 25 mock- (buffer) or virus-infected seedlings per cultivar at 3, 7, and 21 dpi. The exact procedures followed for the isolation, RNA purification, and RNA quality analyses were described previously [32]. Additionally, the absence of RNA contamination was confirmed by performing reverse transcription-polymerase chain reaction (RT-PCR) with *S. tuberosum* elongation factor-1 alpha (*StEf1α* gene) as the reference standard [32]. This test confirmed the absence of contaminating gDNA. Thereafter, cDNA was synthesized using the NG dART RT kit (EURx Sp. z o.o., Gdansk, Poland) according to the recommended protocol. Reverse transcription reactions were performed in a volume of 10 µL using 570 ng of RNA.

### 2.3. Analysis of Expression of PVY^NTN^-NIb and HSC70-8 in Potato Plants Using qPCR

A real-time quantitative polymerase chain reaction (qPCR) was performed using the Bio-Rad CFX96Touch^TM^ apparatus (Bio-Rad Poland Sp. z.o.o., Warsaw, Poland) and SsoAdvanced^TM^ Universal SYBR^®^ Green Supermix (Bio-Rad Polska Sp. z.o.o., Warsaw, Poland) for the reference gene *StHsc70-8*. The TaqMan probe (ACACCAATCTCAACTCCAGATGGAACA) was used to detect the presence of *NIb* with the SensiFAST™ Probe No-ROX Kit (BIOLINE LTD., DNA Gdańsk, Warsaw, Poland), which was compatible with the Bio-Rad CFX96Touch^TM^ apparatus. All the qPCR tests were calibrated with previously prepared five-point calibration curves (based on cDNA and gDNA). Two genes were selected for qPCR analyses, namely the one associated with PVY infection (viral *NIb*, GeneID: KX356068.1) and the host *HSC70-8*. The expression and evaluation of *HSC70-8* (*StHSC70-8,* GeneID: XM_006350698.2) gene in *S. tuberosum* was particularly studied because the complete sequence of the *HSC70* genome was determined and published [39], and the genomic analysis revealed that this protein has highly conservative structural motifs and that its high expression in leaves is strongly associated with pathogenic infections. The expression of PVY^NTN^-*NIb* and *HSC70-8* genes was investigated in both types of potato cultivars. The *S. tuberosum StEf1α* gene (GeneID: AB061263) was used as the reference gene, as suggested in [38]. The primers were designed using Primer3 software (version 0.4.0; Primer3Plus, Free Software Foundation, Inc., Boston, MA, USA). In the case of *StHsc70-8,* we used primers described previously [39]. Table 1 presents all the primers used in the experiments. The starting solution of cDNA (used for the generation of calibration curves) was a fourfold diluted mix of 12 randomly selected cDNA mixes. The calibration curve for gDNA was constructed after an eightfold dilution of the cDNA mix. The remaining subsequent calibration points were measured at fourfold dilutions in a volume of 15 µL. A 5-µL solution of the eightfold-diluted cDNA mix was added to the reaction mixture. The conditions for the qPCR analyses are presented in Table 2 and Table 3.

The expression levels of all the investigated genes and statistical significance as compared to the level of the reference gene *StEf1α* were calculated and normalized, as described previously [39].

### 2.4. Immunofluorescence Localization of NIb and HSC70 in Susceptible and Hypersensitive Potato Leaves

For immunofluorescence localization, leaf samples from mock- and PVY^NTN^-inoculated leaves at 3, 7, and 21 dpi were fixed, embedded in butyl-methyl-methacrylate, and polymerized under ultraviolet light, exactly similar to the method described in [33,34]. Two different primary polyclonal rabbit antibodies were used for each protein. The primary antibody for HSC70 was acquired from Agrisera (Vänäs, Sweden) and used after 1:50 dilution. The second one was designed by GeneCust (Boynes, France) and used for detecting PVY^NTN^-*NIb* (NCBI AQK38487.1) at the region between 2276 and 2794 aa (which includes highly immunogenic C-terminal portion DDELECDTYEVHH) after 1:50 dilution. The primary antibodies were detected using antirabbit IgG antibody conjugated with AlexaFluor488 in 1:100 dilution (Jackson ImmunoResearch Europe Ltd., Cambridgeshire, UK). Olympus UC90 HD camera with Olympus AX70 Provis and UM61002 filter (Olympus, Warsaw, Poland) was used for fluorescence imaging [33,34].

### 2.5. Electron Microscopy, Immunogold Localization, and Statistical Quantification

The leaf samples (mock- and virus-inoculated) of potato plants at 3, 7, and 21 dpi were fixed, embedded in epoxy resin (EPON, Fluka, Switzerland), and polymerized at 60 °C for 24 h as previously described [38]. Then the leaf sections were mounted on Formvar-coated nickel grids and treated according to the procedure of immunogold localization [33,34]. For the localization of *HSC70* and *NIb*, the primary antibodies and dilutions used were similar to those used for immunofluorescent localization (See Section 2.4). The visualizing secondary antirabbit antibodies, conjugated with nanogold particles of size 18 nm, were purchased from Jackson ImmunoResearch Europe Ltd. (Cambridgeshire, UK). The labeling specificity was checked by incubating the grids with samples obtained from mock-inoculated plants and by the omission of the primary antibody from the incubating solution. Immunogold-labeled sections on grids were examined using transmission electron microscopy [33]. After examination, the quantification of protein labeling was done by the method described by Luschin-Ebengreuth and Zechmann [40], and the statistical analyses were performed according to Otulak-Kozieł et al. [33,39]. Specifically, the concentrations of gold particles were analyzed using analysis of variance (ANOVA) and the post hoc Tukey’s HSD test using Statistica software (version 13.0; StatSoft and TIBCO Software Inc., Palo Alto, CA, USA). ANOVA was used as it is an efficient estimator of gold labeling. For the statistical estimation of immunogold labeling, the infected and mock-inoculated materials were compared at 3, 7, and 21 dpi. Gold particles in the cell compartments were counted in 40 fields (10 μm^2^) per image. In each combination (two mock-inoculated plants and PVY^NTN^-inoculated Irys and Sekwana potato plants), gold particles from 200 photos were counted to detect the presence of *HSC70* and *NIb* genes.

### 2.6. Pearson Correlation Coefficient for NIb and HSC70 in Selected Cell Compartments

Based on immunogold quantification results, NIb and HSC70 were found to be localized in three cell compartments: *vacuoles, nuclei,* and chloroplasts. To compare/check the likelihood of occurrence, Pearson’s correlation coefficients (PCCs) were estimated according to Wu et al. [41] and Manders et al. [42] by using Excel 2019 software (Microsoft, Poland, Warsaw). The pairwise correlations between NIb and HSC70 were estimated at the two most significant time points, that is, 3 and 7 dpi, in both potato cultivars. The results were presented in the form of a heat map generated using PCC values, and values over 0.70 *were considered* to reflect the strong positive correlation between both antigens.

### 2.7. Double-Immunogold Localization and Quantification of NIb and HSC70

Double-immunogold localization studies of *PVY*-NIb and HSC70 were performed based on the PCC estimation results. Two sets of antibodies were used for the analysis. The first set consisted of primary and secondary antibodies that were used for the detection of NIb (see Section 2.4) at 1:100 dilution. The second set included a primary mouse monoclonal antibody for detecting HSC70 (Enzo Life Sciences, New York, NY, USA), while the secondary antimouse antibodies were coupled to 10-nm gold particles (Sigma Aldrich, Warsaw, Poland) and used at 1:100 dilution. The embedded plant material (see Section 2.5) was cut into 100-nm sections with an ultramicrotome, placed on nickel grids, and treated as described by Otulak and Garbaczewska and Kozieł et al. [30,43]. Then the grids were treated and labeled using the double-immunogold technique, as previously presented by Kozieł et al. [44]. Double-immunogold colocalization of *NIb* and *HSC70* was analyzed using a 2 × 2 contingency table, as described by Mayhew [45]. Assessments in the 2 × 2 contingency table were performed separately for selected cell structures including vacuoles, nuclei, and chloroplasts using Fisher’s exact test, and the results were computed using GraphPad Software [46]. For more detailed analysis, we also calculated the odds ratio, as described by Mayhew and Lucocq [47].

## 3. Results

### 3.1. PVY^NTN^ Concentration in Susceptible and Hypersensitive Potato Leaves

**Figure 1:**
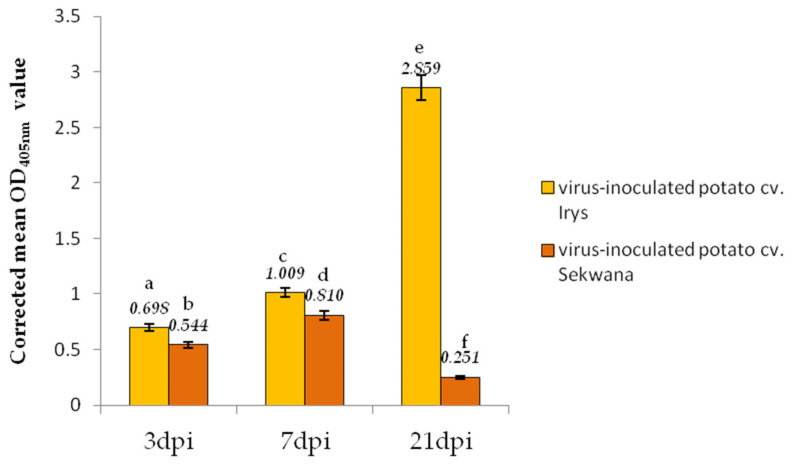
**PVY^NTN^ detection and relative virus concentration** assessment in Irys and Sekwana plants at 3, 7, and 21 dpi. Values represent the mean OD_405nm_ values. The statistical significance of differences was assessed at *p* < 0.05 using ANOVA with post hoc Tukey’s HSD (marked by numbers above the bars).

Irys and Sekwana potato cultivars were used to study the susceptible and resistant interactions with PVY^NTN^, respectively. Sekwana carries the *Ny-1* gene [23] that confers an HR to PVY [24]. The presence of PVY^NTN^ in both the PVY-inoculated cultivars was confirmed by performing a DAS-ELISA assay for the samples collected between 3 and 21 dpi, whereas the virus was not detected in the mock-inoculated plants (Table 1). The OD_405nm_ values were higher in the Irys plants, especially at 7 and 21 dpi, than in the Sekwana plants (Table 4). The corrected mean OD_405nm_ values confirmed a significant increase in the PVY^NTN^ concentrations in Irys cultivar (1.45-fold between 3 and 7 dpi and 2.83-fold between 7 and 21 dpi). In the Sekwana variety, the corrected OD_405nm_ mean values increased 1.49-fold between 3 and 7 dpi but later significantly decreased by 3.23-fold (Figure 1). This observation suggests that in Sekwana the resistance reaction initiated from 7 dpi onward.

### 3.2. Relative Expression of PVY^NTN^-NIb and StHsc70-8 Genes during PVY^NTN^ Infection

**Figure 2:**
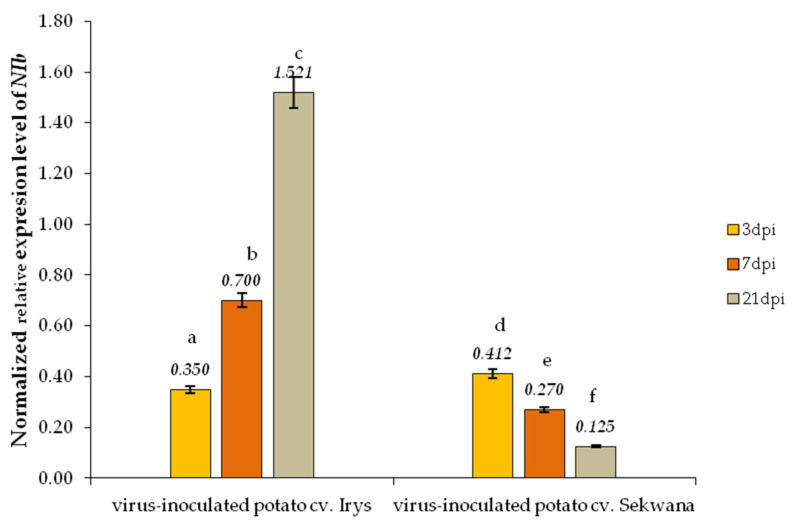
**The normalized relative expression levels of PVY^NTN^-*NIb*** were calculated based on the reference gene *StEf1α* in mock- and virus-inoculated Irys and Sekwana cultivars between 3 and 21 dpi. By using ANOVA and Tukey’s HSD test, the mean values of normalized expression levels were calculated at *p* < 0.05. The statistically significant values are given in numbers above the bars.

**Figure 3:**
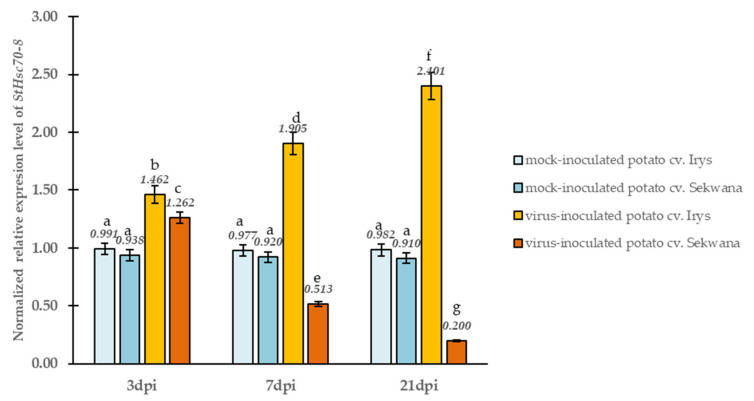
**The normalized relative expression levels of *StHsc70-8*** were calculated based on the reference gene *StEf1α* in mock- and virus-inoculated Irys and Sekwana leaves between 3 and 21 dpi, based on the analyses with ANOVA. Mean values of normalized expression levels were evaluated at *p* < 0.05 and by Tukey’s HSD test. The statistically significant values are given as numbers above the bars.

The expression of the PVY^NTN^-*NIb* and *S. tuberosum Hsc70-8* (*StHsc70-8)* genes is crucial in inducing the viral infection, and they are considered to be essential components for the replication of PVY^NTN^ in the host. The qPCR test was used to determine the expression of these proteins in susceptible and resistant plants. Firstly, the normalized relative expression levels of *NIb* were compared between susceptible and resistance interactions (Irys versus Sekwana, Figure 2). The *NIb* expression was found to be increased (4.35-fold) in Irys from 3 to 21 dpi, but in Sekwana, the expression decreased by 3.30-fold. Interestingly, at the early stages (3 dpi), the rate of *NIb* expression was similar in both the cultivars. A 1.48-fold increase in the normalized relative expression of the *StHsc70-8* gene in Irys and a 1.35-fold increase in Sekwana were observed at 3 dpi when compared to mock-inoculated plants (Figure 3). The expression of *StHsc70-8* continued to escalate at 7 (1.95-fold) and 21 dpi (2.45-fold) in Irys, whereas it decreased at 7 (1.80-fold) and 21 dpi (4.55-fold) in Sekwana. Mock inoculation did not affect the level of expression of *StHsc70-8* in both the cultivars. Furthermore, analysis of the relative virus concentration demonstrated that the highest expression level of both *NIb* and *StHsc70-8* occurred equally in both cultivars at 7 dpi. Surprisingly, this was also the time point when symptoms characteristic for the susceptibility or hypersensitivity were initiated in the plant. This finding suggests that changes in the expression levels were triggered by the cultivar-specific reactions to PVY^NTN^.

### 3.3. PVY^NTN^-NIb Localization Patterns Differ Significantly in Susceptible versus Hypersensitive Leaves

**Figure 4:**
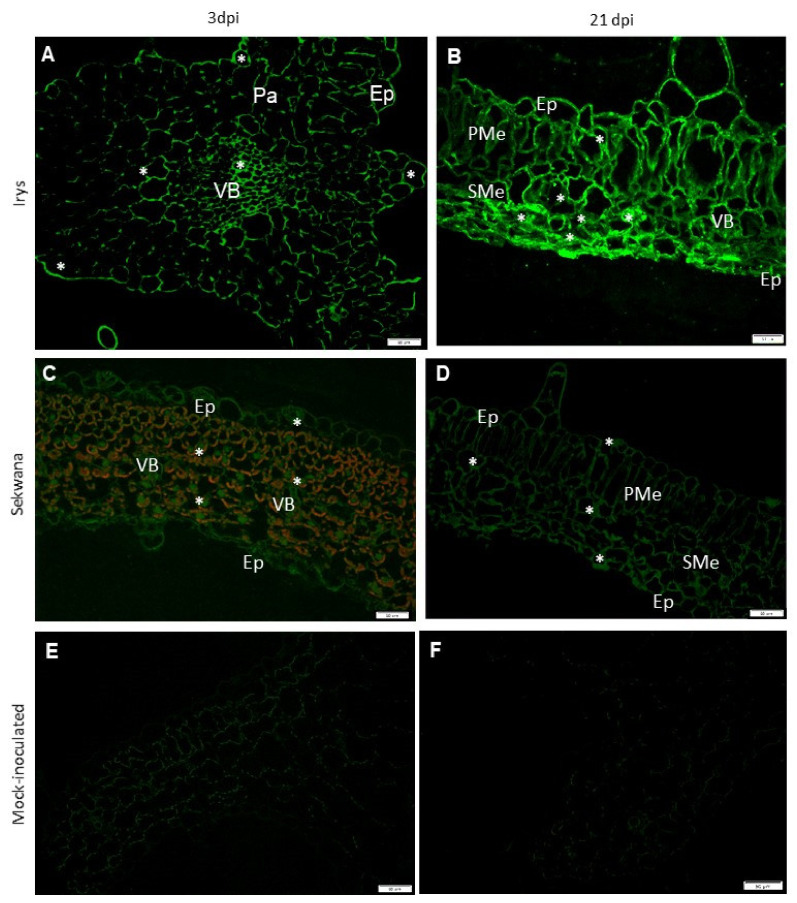
**Localization of PVY^NTN^-NIb by immunofluorescence** in Irys (susceptible: A, B, E) and Sekwana (hypersensitive: C, D, F). (**A**) NIb signal (*) in vascular bundles (VB), parenchyma (Pa), and epidermis (Ep), at 3 dpi in leaf petioles. (**B**) NIb signal (*) predominantly in spongy mesophyll cells (SMe). Signal has been detected in all leaf tissues at 21 dpi. (**C**) NIb signal (*) inside protoplasts of the mesophyll and epidermis in Sekwana at 3 dpi. (**D**) NIb signal mainly in mesophyll cell membranes at 21 dpi in Sekwana. (**E**,**F**) Lack of NIb signal in mock-inoculated Irys (**E**) and Sekwana (**F**), respectively. Scale bar: 50 μm.

The expression patterns of the genes revealed that changes in the expression of proteins occur during the PVY infection in two cultivars. In the next step, PVY^NTN^-Nib replicase and HSC70 proteins (HSC70s) were localized using immunofluorescence and immunogold labeling techniques. The immunolabeling procedure was performed between 3 and 21 dpi and revealed that the occurrence of NIb patterns corresponded with *NIb* gene expression. At 3 dpi, the NIb epitope in Irys mainly localized in the vascular bundles and parenchyma cells (Figure 4A), while a higher NIb concentration was noticed at 21 dpi, particularly in the spongy mesophyll tissue and lower epidermis (Figure 4B). In Sekwana, the NIb fluorescence was observed more predominantly at 3 dpi than at 21 dpi in all the tissues (Figure 4C,D). In Irys, the fluorescence signal at 3 dpi was found to be mainly localized at the membranes, but in Sekwana, the signal was detected in all the protoplasts at 21 dpi. In mock-inoculated plants, no NIb depositions were observed (Figure 4E,F).

**Figure 5:**
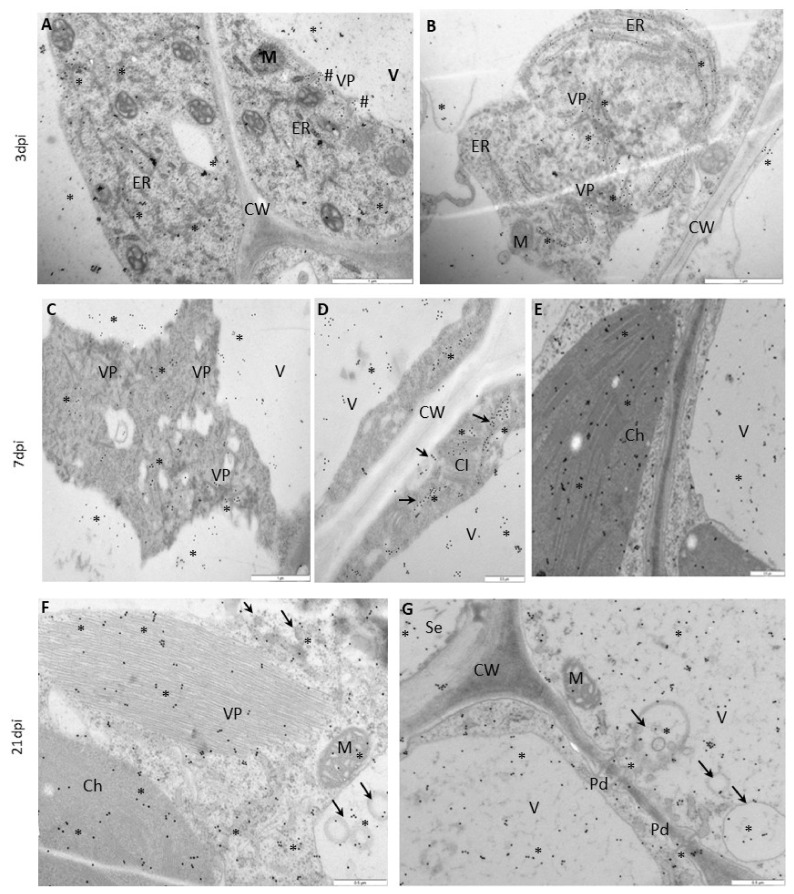
**Immunogold labeling of PVY^NTN^-NIb** in susceptible Irys (**A**,**B**) at 3 dpi, (**C**,**E**) at 7 dpi, and (**F**,**G**) at 21 dpi. (**A**) NIb signal (*) around the ER in the cytoplasm and vacuole (V) of phloem parenchyma cells. Gold granules around virus particles (VP, #) near tonoplasts. Scale bar: 1 μm. (**B**) NIb signal (*) on virus particles (VP) and the ER in mesophyll cells. Scale bar: 1 μm. (**C**) NIb (*) around virus particles (VP) and in the vacuoles (V) of mesophyll cells. Scale bar: 1 μm. (**D**) NIb (*) on membranous structures (arrow), around virus cytoplasmic inclusion (CI) bodies, and in vacuoles (V) in palisade mesophyll cells. Scale bar: 0.5 μm. (**E**) NIb (*) in chloroplasts (Ch) and vacuoles (V) in mesophyll cells. Scale bar: 0.5 μm. (**F**) NIb gold granules (*) in the cytoplasm and virus particles (VP), in chloroplasts (Ch), and vesicular structures (arrows) of cytoplasm and vacuoles (V). Scale bar: 0.5 μm. (**G**) NIb gold granules (*) in plasmodesmata (Pd) and along vesicular structures (arrows) near plasmodesmata. NIb also occurs inside sieve elements (Se) and in the vacuoles of phloem parenchyma cells. Scale bar: 0.5 μm. CW—cell wall, M—mitochondria.

**Figure 6:**
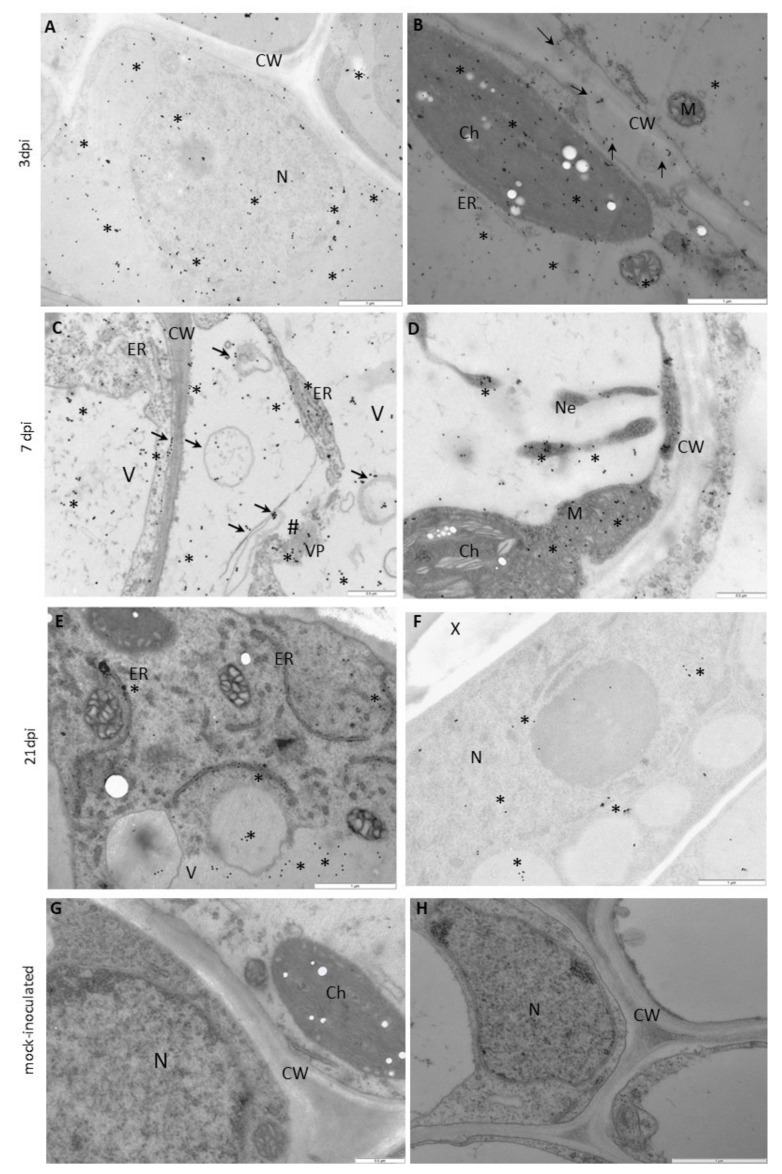
**Immunogold labeling of PVY^NTN^-NIb in Irys and Sekwana leaves:** (**A**,**B**) 3 dpi, (**C**,**D**) 7 dpi, (**E**,**F**) 21 dpi, and (**G**,**H**) mock-inoculated. (**A**) NIb gold deposition (*) in nucleus and cytoplasm in phloem cells. Scale bar: 1 μm (**B**) NIb deposition (*) in chloroplasts (Ch), mitochondria (M), and apoplast areas (arrows). Scale bar: 1 μm. (**C**) NIb (*) in vacuoles (V) and apoplasts with membranous and vesicular structures (arrows). Gold granules also occurred in virus particles (VP, #). Scale bar: 0.5 μm. (**D**) NIb (*) in necrotized protoplasts (Ne) of mesophyll cells. Gold deposition in mitochondria (M) and vacuoles (V). Scale bar: 0.5 μm. (**E**) NIb (*) in vacuoles (V) and along the ER. Scale bar: 1 μm. (**F**) NIb (*) in the nucleus (N) in xylem parenchyma cells. Scale bar: 1 μm. (**G**) Lack of gold deposition in the mesophyll cells of mock-inoculated Irys. Scale bar: 0.5 μm. (**H**) Lack of gold deposition in the phloem of mock-inoculated Sekwana. Scale bar: 1 μm. CW—cell wall, ER—endoplasmic reticulum, X—xylem tracheary elements.

**Figure 7:**
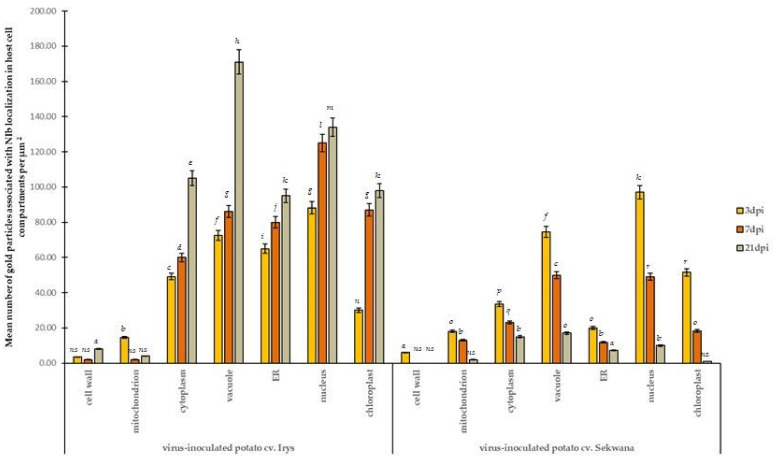
**Statistical significance of PVY^NTN^-NIb immunogold localization in mock- and PVY-inoculated Irys and Sekwana.** The figure presents the mean number of gold particles localized in specific compartments per µm^2^ at 3, 7, and 21 dpi in mock- and virus-inoculated leaves. Quantification of immunogold localization was performed using ANOVA. The mean values were calculated at *p* < 0.05 with the post hoc Tukey’s HSD test. Statistically significant values are marked with letters above the bars. Nonsignificant values are marked as *ns*.

The immunogold labeling procedure was performed to determine the ultrastructural localization of NIb during replication (Figure 5 and Figure 6). The quantification of the NIb epitope revealed characteristic localization patterns and significant differences between Irys and Sekwana. In susceptible Irys, the highest NIb depositions were observed after 3 dpi in the nucleus, vacuole, and ER (Figure 5A,B and Figure 7). Statistically significant increases were observed between 3 and 7 dpi, especially in the chloroplasts and nuclei (Figure 5A–C,E), but the most significant increase occurred between 7 and 21 dpi (Figure 5C–G), with the highest level of deposition in cytoplasm and vacuole (Figure 5F,G and Figure 7). At 21 dpi, NIb deposited around virus particles or within and along the vesicular structures near plasmodesmata, which paralleled the deposition patterns determined by fluorescence analysis. In Irys, gold granules were mainly located in contact with membranous structures at 3 dpi, but at a later stage, they spread into the cytoplasm and were detected in chloroplasts, vacuoles, vesicular structures, and mitochondria (at 7 and 21 dpi).

In hypersensitive Sekwana variety, the immunogold-labeled NIb was localized mainly in the nucleus, vacuoles, and chloroplasts at 3 dpi (Figure 6A,B and Figure 7) and to a lesser extent in vacuoles and membranous structures, but at 7 dpi, it was also found in necrotized mesophyll cells (Figure 6C,D). The statistically significant deposition of NIb decreased between 3 and 21 dpi, with the maximum reduction observed between 7 and 21 dpi (Figure 7). At 21 dpi, NIb was still present in the membranous structures of ER, nucleus, and vacuoles (Figure 6E,F). Generally, at a later stage, the gold particles were found only in vacuoles, nuclei, or in contact with the ER. Apparently, during the ongoing infection, NIb was redistributed from chloroplasts to other selected organelles and membranous structures during the HR. In contrast to this finding, in susceptible potato plants, the concentration of NIb increased in the nucleus and chloroplast. This indicates that the decrease in NIb content and its redistribution to the other organelles during the hypersensitive reaction can be attributed to the plant immune response. The extent of redistribution of NIb and changes in the concentrations of proteins differ in both susceptible and resistant interactions. Later, a progressive decrease in the intensity of NIb deposition was noticed, and at 21 dpi, the protein was restricted to vacuoles and nuclei. No depositions of NIb were found in the mock-inoculated tissues (Figure 6G,H).

### 3.4. Deposition of HSC70 Proteins in Susceptible and Hypersensitive Plants

**Figure 8:**
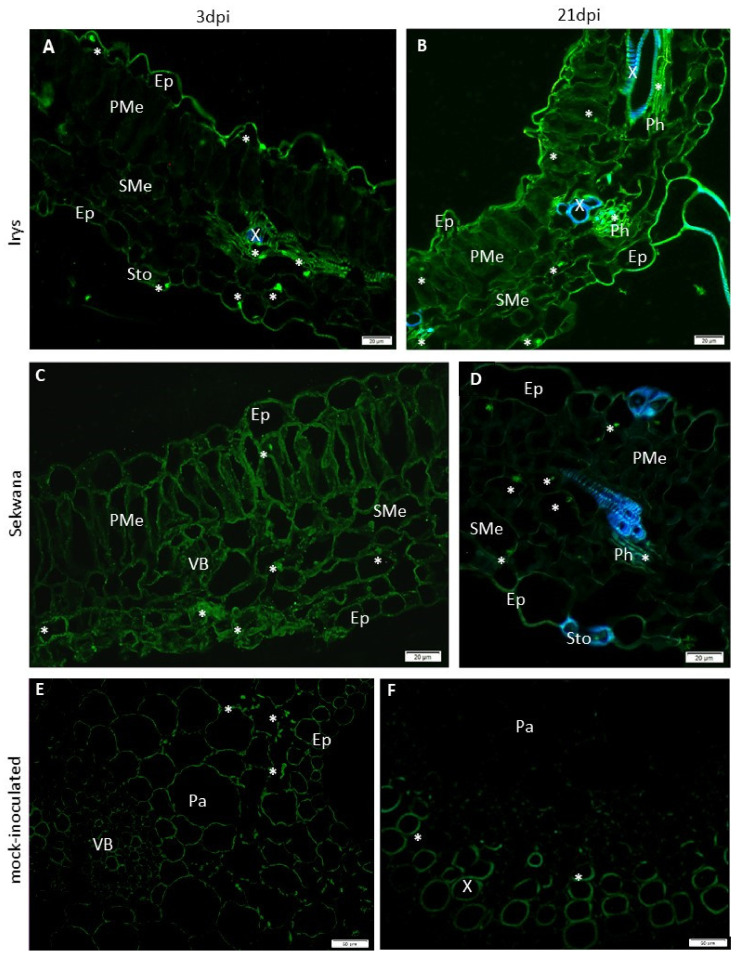
**Immunofluorescence localization of HSC70** in potato leaves of Irys (**A**,**B**,**E**) and Sekwana (**C**,**D**,**F**) inoculated with PVY^NTN^. (**A**) HSC70 (*) points of green fluorescence signal in the epidermis (Ep) and vascular bundle (VB) at 3 dpi. Scale bar: 20 μm. (**B**) HSC70 detection (*) in the inoculated leaf tissue at 21 dpi, mainly in the vascular bundle (VB). Autofluorescence of tracheary elements detected by DAPI. Scale bar: 20 μm. (**C**) HSC70 fluorescence signal (*) in mesophyll at 3 dpi in Sekwana. Scale bar: 20 μm. (**D**) Points of green fluorescence due to HSC70 signal (*) in the spongy mesophyll (SMe) and phloem (Ph) tissues at 21 dpi. Autofluorescence of tracheary elements and cell walls of stomata are detected by DAPI. Scale bar: 20 μm. (**E**) HSC70 (*) in the parenchyma (Pa) and epidermis (Ep) of mock-inoculated Irys. Scale bar: 50 μm. (**F**) HSC70 (*) in xylem tracheary elements (X) in mock-inoculated Sekwana. Scale bar: 50 μm.

The HSC70 proteins are plant host factors that are strongly associated with the potyviral replication complex. Our results reveal that HSC70 proteins are present locally, and the sites of green fluorescence signals indicate that they occur in both the epidermis and vascular bundles in Irys at 3 dpi (Figure 8A). Moreover, at 21 dpi (as compared to 3 dpi), a more intense fluorescence signal indicating the presence of HSC70s in almost all the leaf tissues was noticed in the Irys plant (Figure 8B). A strong HSC70s signal was also detected in all the leaf tissues of the Sekwana plant at 3 dpi (Figure 8C), but only a local, granular green fluorescence was observed in the mesophyll at 21 dpi (Figure 8D). However, similar green fluorescence was also observed locally in the cell wall and membranes of mock-inoculated tissues (Figure 8E,F).

**Figure 9:**
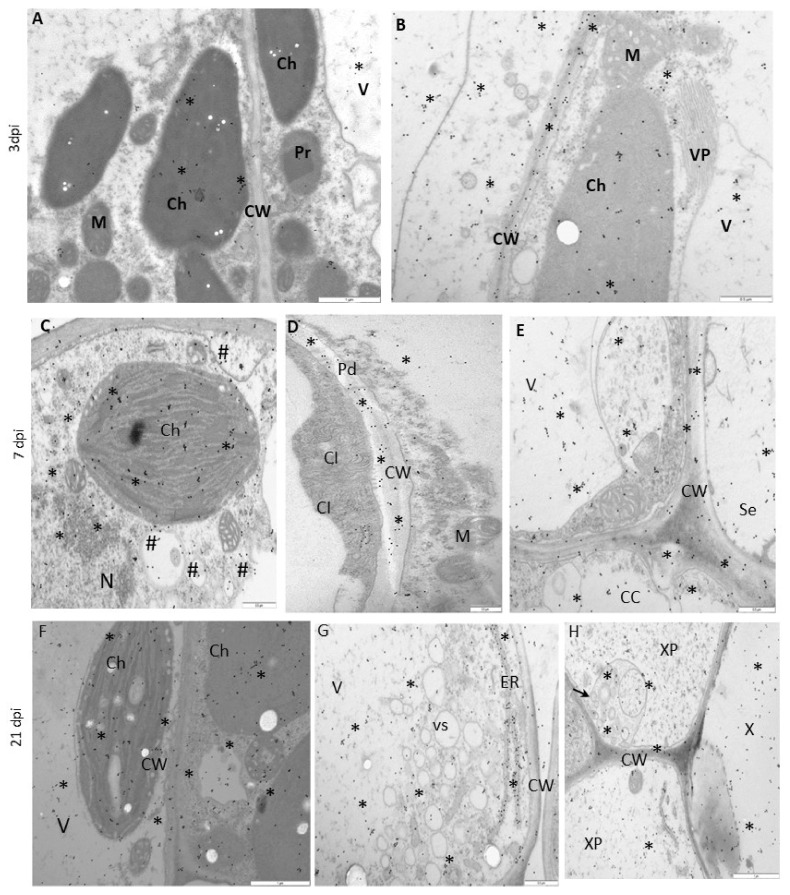
**Immunogold localization of HSC70 in susceptible Irys leaves.** (**A**,**B**) at 3 dpi, (**C**,**E**) at 7 dpi, (**F**,**H**) at 21 dpi. (**A**) HSC70 (*) in chloroplasts (Ch) and vacuoles (V) of mesophyll cells. Scale bar: 1 μm. (**B**) HSC70 (*) in the cell wall and apoplast with vesicles. Gold granules in chloroplasts (Ch) around virus particles (VP) and inside vacuoles (V). Scale bar: 0.5 μm. (**C**) HSC70 (*) in chloroplasts (Ch), nucleus (N), membranous and vesicular structures (#), and in the cytoplasm. Scale bar: 0.5 μm. (**D**) HSC70 (*) in the cell wall (CW) within the plasmodesmata area (Pd) in mesophyll cells. Virus cytoplasmic inclusions (CI) in the cytoplasm. Scale bar: 0.5 μm. (**E**) HSC70 (*) in the sieve element (Se) and companion cells (CC) of the phloem. Gold granules in the cell wall (CW), vacuoles (V), and vesicular and membranous structures. Scale bar: 0.5 μm. (**F**) HSC70 (*) in chloroplasts (Ch), vacuoles (V), and the cytoplasm. Scale bar: 1 μm. (**G**) HSC70 (*) inside and around vesicular structures (vs) and along the ER. Scale bar: 0.5 μm. (**H**) HSC70 (*) in the xylem tracheary element (X) and the cytoplasm of xylem parenchyma cells (XP). Gold granules in multivesicular structures (arrow) and the cell wall (CW). Scale bar: 1 μm. M—mitochondria, Pr—peroxisome.

**Figure 10:**
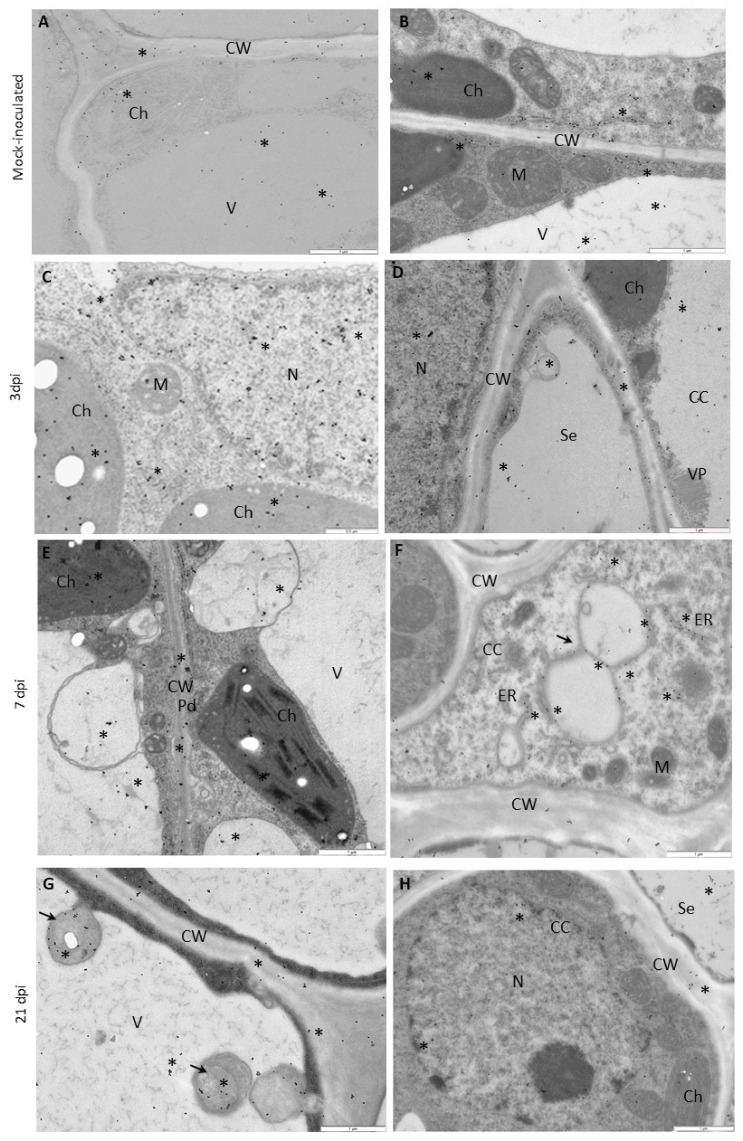
**Immunogold localization of HSC70 in Irys and Sekwana leaves.** (**A**,**B**) Mock-inoculated, (**C**,**D**) at 3 dpi, (**E**,**F**) at 7 dpi, (**G**,**H**) at 21 dpi. (**A**) HSC70 (*) in vacuoles (V), chloroplasts (Ch), and the cell wall (CW) of the phloem of mock-inoculated Irys plants. Scale bar: 1 μm. (**B**) HSC70 (*) along the ER, in vacuoles (V) and chloroplasts (Ch) in the mesophyll of mock-inoculated Sekwana. Scale bar: 1 μm. **(C)** HSC70 (*) in the nucleus (N), chloroplasts (Ch), and cytoplasm in mesophyll cells. Scale bar: 0.5 μm. (**D**) HSC70 (*) in the sieve element (Se) and companion cell (CC). Gold granules in the cell wall (CW), nucleus (N), and vacuoles (V). Virus particles (VP) present in companion cells. Scale bar: 1 μm. **(E)** HSC70 gold granules (*) in vesicular structures, chloroplasts (Ch), and the cell wall (CW) of plasmodesmata (Pd) in mesophyll cells. Scale bar: 1 μm. (**F**) HSC70 gold granules (*) around vesicular structures (arrow) and along the ER in companion cells (CC). Scale bar: 1 μm. (**G**) HSC70 (*) in paramular bodies (arrows), cell wall (CW), and vacuoles (V) of epidermis cells. Scale bar: 1 μm. (**H**) HSC70 (*) in the sieve element (Se) and nucleus (N) in companion cells (CC). Scale bar: 1 μm.

**Figure 11:**
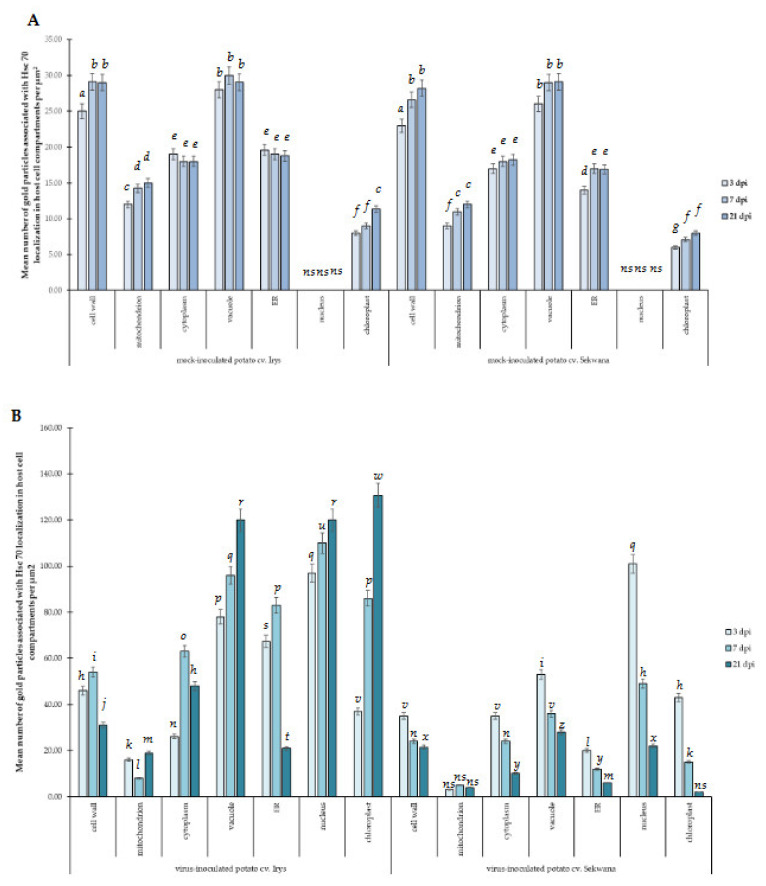
**Statistical significance of HSC70 immunogold localization in host cell compartments** of mock- (**A**) and PVY-inoculated (**B**) potato plants (susceptible Irys and resistant Sekwana). The figure presents the mean numbers of gold particles located in specific compartments per µm^2^ at 3, 7, and 21 dpi in mock- and virus-inoculated plants of both cultivars. Quantification of immunogold localization was performed using ANOVA. The mean values were calculated at *p* < 0.05 using the post hoc Tukey’s HSD test. Statistically significant values at 3, 7, and 21 dpi in mock- or virus-inoculated plants are marked with different letters above the bars. Nonsignificant values are marked as *ns*.

The results of immunogold labeling of the HSC70 proteins were compared between both types of interactions and also against the mock-inoculated plants to specifically analyze the changes in the deposition patterns of HSC70s (Figure 8, Figure 9, Figure 10 and Figure 11). Statistically significant differences between mock- (Figure 11A) and virus-inoculated (Figure 11B) plants were evaluated. The quantification analyses revealed the absence of HSC70 in the nucleus and no major differences between Irys and Sekwana cultivars in the mock-inoculated leaves at both 3 and 21 dpi (Figure 11A,B). In susceptible plants, HSC70 was detected in the mesophyll cells, epidermis, and vascular tissues at 3 dpi (Figure 9A,B). In these plants, the HSC70 protein accumulated mainly in the cell wall within the apoplast region, as well as in the vacuoles and cytoplasm neighboring virus particles (Figure 9B and Figure 11B).

The above-mentioned quantitative localization studies clearly indicated that the induction of HSC70s occurs between 3 and 21 dpi in susceptible Irys plants (Figure 9A–H and Figure 11B) when compared to mock-inoculated Irys (Figure 11A,B). HSC70s were detected in all the leaf tissues of Irys plant, with the highest statistically significant increase observed in chloroplasts, vacuoles, and nuclei (Figure 9F–H and Figure 11B). Conversely, decreased concentrations of the protein were noticed in the cell wall, ER, and the cytoplasm (Figure 11B). In Sekwana, the induction of HSC70s was noticed at 3 dpi (Figure 10A–D and Figure 11A,B), and the highest amounts were detected in the nucleus, vacuoles, and chloroplasts (Figure 11A,B). Interestingly, at 7 (Figure 10C–F) and 21 dpi, the expression of HSC70s decreased in Sekwana (Figure 10E–H). Paramular bodies were observed in the cell walls and vacuoles in Sekwana at 21 dpi. Overall, we observed a statistically significant increase in HSC70 deposition in susceptible Irys at 21 dpi, but it showed a significant decrease in hypersensitive Sekwana, even below the level observed in mock-inoculated Sekwana at 21 dpi. HSC70s were also redistributed during hypersensitive reactions. This finding could suggest that combined redistribution of NIb and HSC70s occurs during HR to infection with PVY, which could be correlated with hypersensitive reaction as an element of activation of plant immune response.

### 3.5. Strong Correlation between PVY-NIb and HSC70 in Chloroplasts, Vacuoles, and Nucleus during Hypersensitive and Susceptible Reactions

**Figure 12:**
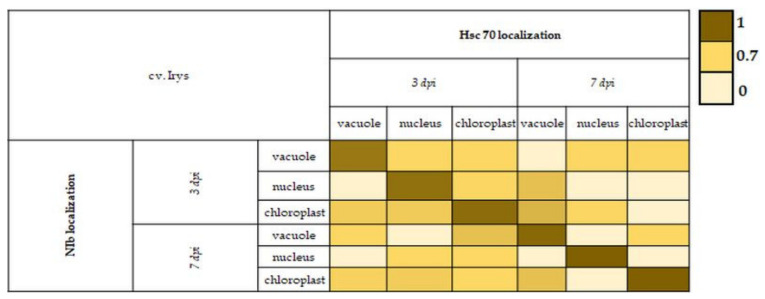
Heatmap of PCC **for NIb and HSC70 localization in** virus-inoculated **Irys from 3 to 7 dpi.** PCC matrix values are presented pairwise for specific cell compartments in specific time dpi and marked with colors, from very dark yellow (PCC = 1) to bright yellow (PCC = 0).

**Figure 13:**
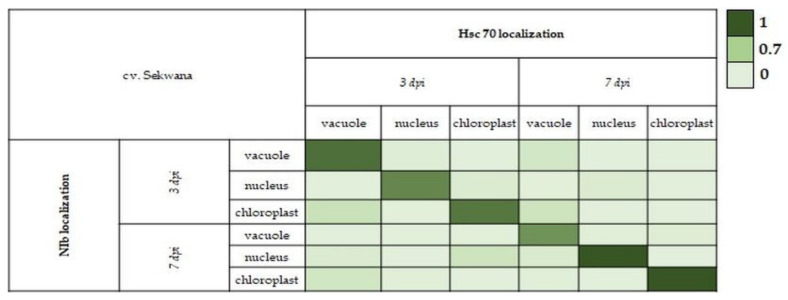
Heat map of PCC **for PVY-NIb and HSC70 localization in** virus-inoculated Sekwana **from 3 to 7 dpi.** PCC values are presented pairwise for specific cell compartments at a specific time after virus inoculation and marked with colors from very dark green (PCC = 1) to pale green (PCC = 0).

Further quantification of NIb and HSC70 localizations by single-immunogold labeling suggested that both proteins (in both cultivars) appeared frequently in similar cell compartments including vacuoles, nuclei, *and chloroplasts*. The results also showed that some amount of redistribution takes place during HR. Therefore, we attempted to evaluate if there was any correlation between these depositions by calculating the PCC separately for Irys (Figure 12) and Sekwana (Figure 13) varieties at 3 and 7 dpi (the time points showing the most significant depositions).

The statistical analyses showed that (Figure 12) the increase in PVY-NIb was positively correlated with the increase in HSC70 in specific cell compartments at 3 and 7 dpi in Irys cultivar. The PCC values were higher at 7 dpi (0.94–1.00) than at 3 dpi (0.80–0.90), which suggested that the correlation increased along with the time of infection. However, in Sekwana the correlation decreased (Figure 13), suggesting that a decrease in NIb reduced HSC70 (or vice versa) at 3 and 7 dpi. It also indicated that changes in NIb are associated with redistribution of HSC70. Generally, the PCC values in Sekwana were slightly higher than those found in Irys. In the resistant cultivar, the increase in PCC values between 3 and 7 dpi was not noted.

### 3.6. Colocalization of PVY-NIb and HSC70 in PVY^NTN^-Infected Leaves

To confirm that both these proteins colocalized in susceptible and resistant cultivars, double-immunogold assays were performed between 3 and 7 dpi. Correlation analyses showed a statistically significant association between NIb and HSC70 in certain cell compartments of both cultivars. However, this showed only an indirect association between the concentrations of both proteins. To confirm colocalization directly at the ultrastructural level, the double-immunogold localization technique was performed. In susceptible Irys, both epitopes were co-detected inside the vacuoles, nuclei, chloroplasts, and cytoplasm at 3 (Figure S1A) and 7 (Figure S1B) dpi. Colocalization was found to be increased between 3 and 7 dpi in Irys, which corresponded with the localization results obtained by single immunogold labeling. In general, the amount of colocalized proteins was higher in Irys than in resistant Sekwana, but the colocalized regions were almost similar for both cultivars. Both epitopes were copresent in vacuoles, nuclei, and chloroplasts at 3 (Figure S1C) and 7 dpi (Figure S1D). Similar to single-immunogold assays, the expression of NIb and HSC70 decreased between 3 and 7 dpi, which has been statistically quantified by dual-labeling analysis in the selected cell compartments (Table S1A–D). The odds ratio (OR) values and the results of Fisher’s test for double-immunogold parameters (estimated at statistical values *p* < 0.001) revealed that the zero hypothesis, which proposed that localizations of PVY-NIb and HSC70 were independent of each other, has to be reconsidered. Positive values of the OR in vacuoles, nucleus, and chloroplast at 3 and 7 dpi in potato Irys (Table S1A,B) and Sekwana (Table S1C,D) plants, accompanied by a high incidence of double positives, indicated the statistically significant colocalization of both proteins (Table S1A–D). In addition, OR values showed that the strongest colocalization of NIb and HSC70 occurred in the nucleus (OR = 76.3) and in vacuoles (OR = 50) at 7 dpi in Irys (Table S1B). However, Sekwana showed the lowest values (Table S1D) for vacuoles (OR = 6) and chloroplasts (OR = 4.33) at 7 dpi.

## 4. Discussion

Similar to other viruses, PVY can modify host cell machinery to successfully “direct” and establish the infection cycle. The interaction between viruses and plants involves complex mechanisms, such as exploiting host factors needed for efficient viral infection and replication process, and conversely, mechanisms of plant resistance to viruses by activating various defense pathways. Viral proteins are involved in most of the steps of virus infection, including replication, translation, and local or systemic transport [48,49]. Many proteins are multifunctional and participate in multiple stages of dynamic interactions among RNA, viral proteins, and host proteins [50,51]. The type of interaction is dependent on the plant genotype, environmental conditions, and virus strain.

Potato cultivars have different genetic profiles and stimulate different types of responses [52]. It has been postulated that the plant immune system plays a key role in the front-line defense against pathogens and participates in the recognition of invader and pathogen-associated molecular patterns (PAMPs), for example, the membranous PRRs [53]. However, PAMPs trigger immunity, known as PTI, and can potentially act against viruses [54,55,56], but the sensing mechanism of PTI is unknown [57]. The second potential “front-line” defense in the plant immune system is provided effector-triggered immunity. In this case, the pathogen effectors can be recognized by receptors and resistance protein R, which results in disease resistance [53].

During incompatible potato interactions, the defense response restricts virus multiplication and transport [30,58,59]. One of the methods adopted by plants to develop resistance against PVY is by the activation of HR, which is manifested by the formation of local necrotic lesions on the inoculated leaves. In potato plants, dominant R genes provide two types of resistance against PVY: extreme resistance conferred by *R* genes and HR (conferred by *Ny* genes) [60]. Therefore, our comparative studies focused on testing the changes/modulation in the PVY-*Nib* replicase and host *StHSC70-8* gene expression, as well as the spatial distribution of the PVY-NIb and HSC70 proteins, in two potato cultivars that responded differently to PVY^NTN^ inoculation. The cultivar Irys revealed a susceptible reaction (resistance level of 5.5 on a scale of 1–9) [27], but cultivar Sekwana, carrying the *Ny-1* gene on chromosome IX, conferred an HR to PVY infection (resistance level of 8 on a 1–9 scale) [26,27]. A hypersensitive reaction was observed after 6–7 days on inoculated PVY leaves, whereas a susceptible systemic response was observed at 10–15 dpi. Moreover, we confirmed that in susceptible Irys, relative virus accumulation gradually increased from 0 to 21 dpi. On the contrary, in hypersensitive Sekwana, the relative virus titer slightly increased from 0 to 7 dpi, followed by a steady decrease between 7 and 21 dpi. Based on these results, we postulate that the seventh day is a crucial time point post-inoculation, as the host starts to restrict the relative virus accumulation by inducing an HR.

One of the eleven known PVY-encoded proteins is the NIb, which is an RdRp required for PVY RNA replication [61,62] by the membrane-bound viral replication complex (VRC) [63,64]. The structural conformation of RdRp displays a right hand with three functional domains [65], which include five conservative motifs A–E [66]. The GDD is located in the C motif and is crucial for RdRp activity, as demonstrated by Li and Carrington [67] where substitutions in GDD or the NIa/NIb cleavage site were lethal to tobacco protoplasts. The finger subdomain contains two conserved motifs F and G [68]. The potyviral NIb carries all the seven conserved motifs [69], with motifs A, B, D, and E recognizing/binding the nucleoside triphosphates, while A and G bind the metal cations and are involved in the transfer of the phosphate groups. Motif D holds the structural integrity, E primes the nucleotide-binding, while B, F, and G bind the RNA template [70].

In this work, we investigated the pattern of PVY^NTN^*-NIb* gene expression and the in situ localization of the 15-aminoacid-long C-terminal portion of PVY^NTN^-*NIb* in susceptible versus hypersensitive potato hosts. The expression of the PVY^NTN^*-NIb* gene, as studied by qPCR, initially increased in both cultivars, especially between 7 and 21 dpi in Irys, but steadily decreased in hypersensitive Sekwana. This finding indicates that PVY^NTN^*-NIb* functions as an active component in plant–virus susceptible interactions [71,72]. Up to 3 dpi, the PVY^NTN^*-NIb* gene was weakly induced in Sekwana, which suggests that *PVY^NTN^-NIb* plays a potential role in signaling the resistance response. Virus infection is usually restricted if the host is capable of eliciting an effective antiviral defense response [73].

Previous results, consistent with the association between RdRp-NIb and plant cell death, showed that resistance reactions in tobacco involve autophagy processes. Janzac et al. [74,75] demonstrated that the CM334 variety contains a single dominant resistance gene *Pvr4* which confers dominant resistance to several potyviruses, including PVY in pepper plants. The protein encoded by *Pvr4* has been found to recognize the virus NIb, thus initiating the resistance mechanism. In addition, Kim et al. [76] suggested that the interaction between NIb and the LRR domain of Pvr4 suppresses the CC domain and activates a cell death reaction. According to the authors, NIb may function as ETI to inhibit virus infection. Along these lines, PVY-NIb strain MsNr was considered to be an elicitor of the HR in tobacco plants that carried the R3 gene, which confers resistance to nematodes, as postulated by Fellers et al. [77].

The results of another study that demonstrates the relationship between NIb and various types of plant cell death has been presented by Li et al. [78]. The authors surmise that a core component of autophagy genes (ATG 6), known as Beclin-1, can interact with NIb of turnip mosaic virus (TuMV), which then degrades NIb in autophagosomes. Our data indicate that NIb is a key component in the virus infection cycle and drives virus replication and multiplication, but probably this is not the only function performed by NIb. While analyzing the spatial distribution of the C-terminal portion of NIb during PVY^NTN^-hypersensitive and PVY^NTN^-susceptible interactions up to 21 dpi, maximum NIb deposition was observed at 3 dpi, regardless of the type of interaction. But thereafter, NIb gradually increased in Irys whereas decreased significantly in Sekwana. The PVY^NTN^-NIb protein is predominantly localized in the nucleus, vacuoles, and the ER, and a drastic increase was noticed in vacuoles, nucleus, and chloroplasts. However, a dynamic withdrawal of NIb from the nucleus was observed in Sekwana between 3 and 21 dpi. This kind of viral replicase redistribution seems to be a “symptom” that indicates the activation of the plant immune response. The increased mobility into the nucleus during susceptible host interaction was also observed by Zhang et al. [79] during the infection of *Arabidopsis thaliana* and *Nicotiana benthamiana* with TuMV. The increased TuMV-NIb nuclear mobility was associated with the sumoylation (small ubiquitin-like modifier) pathway [79]. This pathway enabled the mobility of NIb into the nucleus. Zhang et al. [79] suggested that some amount of TuMV-NIb is also transported again from the nucleus to the cytoplasm by exportin 1 (XPO1) protein. Mutation in xpo1 inhibited the TuMV infection. However, when the investigations of TuMV were conducted in the plants with no HR genes, the presence of XPO1 in potatoes was not confirmed. In this context, it seems that during HR in potatoes, there is a constant decrease in the concentration of PVY^NTN^-NIb in the nucleus. This reaction pattern is also supported by the observation that HSC70 is withdrawn in the HR reaction. Furthermore, it has been confirmed previously that PVY particles are localized inside the cell organelles and that they are mainly attached to the nuclear envelope of chloroplasts during the susceptible interactions [30,80]. This indicated that PVY^NTN^ multiplies in these organelles, possibly by transforming membranes into vesicles that host the replication complex [81]. In addition, TuMV potyvirus utilizes 6K2-VPg to induce the formation of vesicular structures from the trans-Golgi network and the periphery of chloroplasts [82], suggesting a similar mechanism for PVY [83,84]. Movahed et al. [82] reported that TuMV replication complexes could be transported within the specific type of vesicles into the extracellular matrix. Similar to the ultrastructural observations of PPV-NIb [13], we detected PVY^NTN^-NIb around virus particles in the cytoplasm and in the cytoplasmic inclusion bodies, which are found in vesicles. In addition, Martín et al. [84] and Riedel et al. [13] localized the tobacco etch virus NIb (TEV-NIb) in *Nicotiana tabacum* Samsun NN and PPV-NIb in *Nicotiana clevelandii* in the crystalline nuclear inclusions by using silver-enhanced IgG gold conjugates. Our ultrastructural analyses revealed the presence of PVY^NTN^-NIb in the vascular tissue, plasmodesmata, and vesicular structures that contact the plasmodesmata in the susceptible Irys.

Our previous studies concentrated on changes that take place in the cell wall metabolism during PVY–potato interactions and underlined the role of expansins in loosening the cell walls during susceptible responses [32]. Simultaneous induction of *StEXPA3* [32] and PVY^NTN^*-NIb* gene expression, together with the increased deposition of expansins, might occur within the same locations and these proteins perform molecular functions together, as already postulated by Wei et al. [64] and Li et al. [85]. The authors suggested an interaction between EXPA1 and TuMV-NIb in *N. benthamiana*, where NbEXPA1-NIb reduced the level of *NbEXPA1* in the plasmodesmata, leading to antiviral defense. However, the overexpression of *NbEXPA1* promoted TuMV replication and local mobility of the virus [86].

NIb, in addition to its role in the virus infection cycle, also performs other functions by localizing with some host proteins, likely activating the VRC. More studies are needed to explore the underlying mechanisms of PVY replication. The NIb may also participate in early defense reactions, which could be confirmed by the analysis of target host factors that cooperate with the PVY-NIb replicase complex.

Many host proteins change their location during viral infection. Cytoplasmic proteins can move to the sites of virus multiplication, such as mitochondria, chloroplasts, or nuclei. They can participate in essential processes such as the formation of sites of virus replication or stabilization of viral RdRp [87,88]. HSPs (HSP family) belong to such a group. Plant HSPs are involved in many physiological functions, especially during abiotic and biotic interactions [22,23]. HSPs were found to be recruited to virus replication sites [16,63]. Dufresne et al. [16] and Hafren et al. [48] confirmed the presence of HSC70 in the replication complex of TuMV and PVA potyviruses. Therefore, in this study, we explored the expression of the *StHSC70-8* gene, which showed that high levels of HSC70 protein were produced during both hypersensitive and susceptible reactions. The expression of HSC70 gradually increased in susceptible reactions up to 21 dpi but decreased in Sekwana after 3 dpi. These findings are in contrast to those published by Makarowa et al. [89], which revealed that PVY infection did not activate the expression of *hsp* genes at 22 °C. However, the authors tested different PVY strains in different potato cultivars and at different temperatures and SA-treated conditions. They postulated that PVY^0^ exerted no notable effects on the heat–stress response in resistant plants, whereas in susceptible hosts, PVY^0^ infection induced the HSP signaling pathway and modulated the HSP response triggered by heat stress. Hýsková et al. [90] observed that the application of heat shock before or after inoculation of the Petit Havana tobacco with PVY accelerated the propagation of infection.

Another group of viruses showed similar observations for susceptible and resistant PVY^NTN^–potato interactions. HSP70 enhanced the rate of infection in *N. benthamiana* with tobacco mosaic virus, potato virus X, and watermelon mosaic virus [48,91,92], similar to that observed in susceptible Irys with PVY^NTN^. On the other hand, a decrease in HSC70 levels decreased the accumulation of viral particles, leading to an HR in Sekwana. These trends were observed not only for plant–virus interactions but also for other pathogens. Regulation of HSP70 expression was analogous to the HSP70-regulated immune response in *A. thaliana* [93]. HSP70 is required for the HR to *Phytophtora infestans* and non-host resistance to *Pseudomonas cichorii* in *N. benthamiana* [93]. Downregulation of HSP70 reduced the accumulation of TYLCV [94], whereas downregulation of both HSP70 and HSP90 affected the infection by red clover necrotic mosaic virus (RCNMV) [62,95].

The above-mentioned results showed that parallel to *StHSC70-8* gene expression, deposition of HSC70 gradually increased up to 21 dpi in susceptible Irys. Thus, the virus infection must be responsible for the elevated HSC70 depositions. Interestingly, HSC70 accumulated predominantly in nuclei, and to a lesser extent in chloroplasts. We conclude that HSC70 can participate in defense response signaling and cooperate with RdRp-Nib in the host. However, during HR at 3 dpi, HSC70 and PVY^NTN^-NIb also colocalize in the same compartments. Similar results have been reported by Wang et al. [96] about the association of HSC70-2 protein with the beet black scorch virus (BBSV) replication protein p23. The authors demonstrated that the interaction of HSC70-2 with BBSV capsid protein and p23 was essential for BBSV replication [96]. Our findings of colocalization of HSC70 and PVY-NIb in the same compartments suggest that HSC70 might be an active component of the replication complex, but can also be involved in systemic signaling during infection.

HSC70 can be relocated from cytoplasm and ER into nuclei, chloroplasts, or vacuoles. Gorovits et al. [94] suggested that HSP70 moves from the cytoplasm to the nucleus during TYLCV infection. However, Dufresne et al. [16] noticed translocation to the ER membranes during TuMV infection. Moreover, HSP70 and HSP90 localized mainly in the cytoplasm, but inoculation with Red clover necrotic mosaic virus (RCNMV revealed that both proteins accumulated mostly in the ER [95]. HSP90 enhanced viral propagation and was detected in chloroplasts, thus playing an important role in the *Bamboo mosaic virus* replication process [97]. Our findings confirm that the strongest colocalization of PVY^NTN^-NIb and HSC70 is observed in the nucleus and vacuoles at 7 dpi in susceptible Irys, but the lowest in hypersensitive Sekwana. Therefore, the distribution of the HSC protein family can change dynamically during different plant–virus interactions, depending on the type of interaction, virus group, and the site of virus multiplication in the cell. Moreover, the HSC70 mobility and changes in its levels strongly correlate with NIb expression during infection.

## 5. Conclusions

Our findings of the expression and colocalization of HSC70 and PVY^NTN^-NIb in the same cellular compartments during compatible (susceptible) and incompatible (hypersensitive) reactions strongly suggest their correlation/modulation during plant response to PVY infection. It is most likely that these two proteins cooperate at some stages during the replication cycle. It is a well-known fact that viral accumulation can trigger changes in the plant gene expression and subcellular localization of certain plant proteins, which could further enhance or restrict virus accumulation. Antiviral defense can be mediated by host factors that affect virus replication and movement. However, viruses can counterattack by suppressing the activity of antiviral defense components produced by plants. Taken together, our findings reveal that StHSC70 and PVY^NTN^-NIb likely manifest their activities as team players, thus affecting PVY replication and accumulation during susceptible infection and possibly participating in systemic signaling of plant–virus interactions. Moreover, the absence of correlated redistribution of NIb and StHSC70 from some regions of the cell during viral infection increases the concentration of both proteins in cell compartments, which is associated with susceptibility. However, during HR, HSC70 may initially cooperate with PVY^NTN^ but at later points can stimulate the defense response signaling via interaction with other defense factors. This requires the redistribution of NIb and StHSC70 proteins from the selected cell compartments (especially the nucleus), which promotes the increased reduction of these proteins during resistance response. Further studies are needed to identify the host pathways that actively impact NIb–HSC70 contribution during PVY^NTN^–potato interaction.

## Figures and Tables

**Table 1:** Gene accession numbers, primer sequences, and product lengths for RT-qPCR analyses.

Genes	*Gene ID*	Forward Primer	Reverse Primer	Temperature of Primer Annealing (°C)	Concentration in Reaction (μM)	Product Length (bp)
**Investigated**						
*StHsc70-8*	XM_006350698.2	5′-AGGGATGCCAAGATGGACAA-3′	3′-AACAGCTCATCTGGGTTGA-5′	58	0.5	144
*Nib*	KX356068.1	5′-CTCATCATCAGAAGCACATACA-3′	3′-GCATAGCAAGGACAACCAT-5′	58	0.4	190
**Reference**						
*StEF1a*	AB061263	5′-GGTGATGCTGGTATGGTTAAG-3′	3′-GGTCCTTCTTGTCAACATTCTT-5′	58	0.5	148

**Table 2:** Conditions of the RT-qPCR for the reference genes (*).

Program	Parameters
Preliminary denaturation	95 °C for 5 min
Amplification (35 cycles)	95 °C for 10 s 58 °C for 10 s 72 °C for 20 s *
Melting curve	65–95 °C; 0.1 °C/s

* Fluorescence signal reading was taken at the final stage.

**Table 3:** Conditions of the qPCR analysis for the *NIb* ORF (*).

Program	Parameters
Preliminary denaturation	95 °C for 10 min
Amplification (35 cycles)	95 °C for 10 s 58 °C for 10 s *

* Fluorescence signal reading was taken at the final stage.

**Table 4:** PVY^NTN^ detection using DAS-ELISA assay in mock- and virus-inoculated potato plants as reflected by mean OD_405nm_ values. The absence of a virus is marked by (−). The presence of PVY^NTN^ (**+**) is considered positive when the mean OD_405nm_ values reach the cut-off point (0.135).

Sample	Mean OD_450nm_	Presence (+)/Absence of the Virus (−)
Buffer	0.0000	−
Mock-inoculated potato cv. Irys (3 dpi)	*0.0403*	−
Mock-inoculated potato cv. Sekwana (3 dpi)	*0.0399*	−
PVY^NTN^-inoculated potato cv. Irys (3 dpi)	*0.7383*	+
PVY^NTN^-inoculated potato cv. Sekwana (3 dpi)	*0.5839*	+
Mock-inoculated potato cv. Irys (7 dpi)	*0.0408*	−
Mock-inoculated potato cv. Sekwana (7 dpi)	*0.0404*	−
PVY^NTN^-inoculated potato cv. Irys (7 dpi)	*1.0498*	+
PVY^NTN^-inoculated potato cv. Sekwana (7 dpi)	*0.8504*	+
Mock-inoculated potato cv. Irys (21 dpi)	*0.0540*	−
Mock-inoculated potato cv. Sekwana (21 dpi)	*0.0551*	−
PVY^NTN^-inoculated potato cv. Irys (21 dpi)	*2.913*	+
PVY^NTN^-inoculated potato cv. Sekwana (21 dpi)	*0.3061*	+

## Data Availability

Excluded.

## Supplementary Materials

The following text is available online at https://www.mdpi.com/article/10.3390/vaccines9111254/s1. Figure S1: Double-immunogold labeling of PVY-NIb (black arrowhead) and HSC70 (white arrowhead) in susceptible (A and B) and resistant (C and D) PVY^NTN^–potato interactions. Table S1: Quantification of preferential double-immunogold localization of PVY-NIb and HSC70 in 2 × 2 contingency table from GraphPad Software.

## References

[1] Rybicki E.P. (2015). A top ten list for economically important plant viruses. Arch. Virol..

[2] Scholthof K.B.G., Adkins S., Czosnek H., Palukaitis P., Jacquot E., Hohn T., Hohn B., Saunders K., Candresse T., Ahlquist P. (2011). Top 10 plant viruses in molecular plant pathology. Mol. Plant. Pathol..

[3] Chung B.Y., Miller W.A., Atkins J.F., Firth A.E. (2008). An overlapping essential gene in the Potyviridae. Proc. Natl. Acad. Sci. USA.

[4] Revers F., García J.A. (2015). Molecular biology of potyviruses. Adv. Virus Res..

[5] Olspert A., Chung B.Y., Atkins J.F., Carr J.P., Firth A.E. (2015). Transcriptional slippage in the positive-sense RNA virus family Potyviridae. EMBO Rep..

[6] Rodamilans B., Valli A., Mingot A., San León D., Baulcombe D., López-Moya J.J., García J.A. (2015). RNA polymerase slippage as a mechanism for the production of frameshift gene products in plant viruses of the *Potyviridae* family. J. Virol..

[7] Cordeo T., Mohamed M.A., López-Moya J.J., Daròs J.A. (2017). A Recombinant *Potato virus Y* infectious clone Tagged with the Rosea1 Visual Marker (PVY-Ros1) facilitates the analysis of viral Infectivity and allows the production of large amounts of anthocyanins in plants. Front. Microbiol..

[8] Cui H., Wang A. (2019). The biological impact of the hypervariable N-terminal region of potyviral genomes. Annu. Rev. Virol..

[9] Acosta-Leal R., Xiong X. (2008). Complementary functions of two recessive R-genes determine resistance durability of tobacco ‘Virgin A Mutant’ (VAM) to *Potato virus Y*. Virology.

[10] Martín M.T., García J.A. (1991). Plum pox potyvirus RNA replication in a crude membrane fraction from infected *Nicotiana clevelandii* leaves. J. Gen. Virol..

[11] Schaad M.C., Jensen P.E., Carrington J.C. (1997). Formation of plant RNA virus replication complexes on membranes: Role of an endoplasmic reticulum-targeted viral protein. EMBO J..

[12] Léonard S., Viel C., Beauchemin C., Daigneault N. (2004). Interaction of VPg-Pro of Turnip mosaic virus with the translation initiation factor 4E and the poly(A)-binding protein in planta. J. Gen. Virol..

[13] Riedel D., Lesemann D.E., Maiss E. (1998). Ultrastructural localization of nonstructural and coat proteins of 19 potyviruses using antisera to bacterially expressed proteins of plum pox potyvirus. Arch. Virol..

[14] Wang X., Ullah Z., Grumet R. (2000). Interaction between Zucchini yellow mosaic potyvirus RNA-dependent RNA polymerase and host poly-(A) binding protein. Virology.

[15] Thivierge K., Cotton S., Ufresne P.J.D., Mathieu I., Beauchemin C., Ide C., Fortin M.G., Laliberté J.F. (2008). Eukaryotic elongation factor 1A interacts with Turnip mosaic virus RNA-dependent RNA polymerase and VPg-Pro in virus-induced vesicles. Virology.

[16] Dufresne P.J., Thivierge K., Cotton S., Beauchemin C., Ide C., Ubalijoro E., Laliberté J.F., Fortin M.G. (2008). Heat shock 70 protein interaction with Turnip mosaic virus RNA-dependent RNA polymerase within virus-induced membrane vesicles. Virology.

[17] Park C.J., Seo Y.S. (2015). Heat shock proteins: A review of the molecular chaperones for plant immunity. Plant. Pathol. J..

[18] Huttner S., Strasser R. (2012). Endoplasmic reticulum-associated degradation of glycoproteins in plants. Front. Plant. Sci..

[19] Whitley D., Goldberg S.P., Jordan W.D. (1999). Heat shock proteins: A review of the molecular chaperones. J. Vasc. Sur..

[20] Gupta S.C., Sharma A., Mishra M., Mishra R.K., Chowdhuri D.K. (2010). Heat shock proteins in toxicology: How close and how far?. Life Sci..

[21] Gupta D., Tuteja N. (2011). Chaperones and foldases in endoplasmic reticulum stress signaling in plants. Plant. Signal. Behav..

[22] Usman M.G., Rafii M.Y., Martini M.Y., Yusuff O.A., Ismail M.R., Miah G. (2017). Molecular analysis of Hsp70 mechanisms in plants and their function in response to stress. Biotechnol. Genet. Eng. Rev..

[23] Anaraki Z.E., Hosseini A., Shariati M. (2018). Transient silencing of heat shock proteins showed remarkable roles for HSP70 during adaptation to stress in plants. Environ. Exp. Bot..

[24] Ul Haq S., Khan A., Ali M., Khattak A.M., Gai W.-X., Zhang H.-X., Wei A.-M., Gong Z.-H. (2019). Heat shock proteins: Dynamic biomolecules to counter plant biotic and abiotic stresses. Int. J. Mol. Sci..

[25] Bolhassani A., Agi E. (2019). Heat shock proteins in infection. Clin. Chim. Acta.

[26] Szajko K., Strzelczyk-Żyta D., Marczewski W. (2014). *Ny*-*1* and *Ny*-*2* genes conferring hypersensitive response to *Potato virus Y* (PVY) in cultivated potatoes: Mapping and marker-assisted selection validation for PVY resistance in potato breeding. Mol. Breed..

[27] Zimoch-Guzowska E., Yin Z., Chrzanowska M., Flis B. (2013). Sources and effectiveness of potato PVY resistance in IHAR’s breeding research. Am. J. Potato Res..

[28] The European Cultivated Potato Database. https://www.europotato.org/quick_search.php.

[29] Szajko K., Chrzanowska M., Witek K., Strzelczyk-Żyta D., Zagórska H., Gebhart C., Hennig J., Marczewski W. (2008). The novel gene Ny-1 on potato chromosome IX confers hypersensitive resistance to *Potato virus Y* and is an alternative to Ry genes in potato breeding for PVY resistance. Theor. Appl. Genes.

[30] Otulak K., Garbaczewska G. (2010). Ultrastructural events during hypersensitive response of potato cv. Rywal infected with necrotic strains of *Potato virus Y*. Acta Physiol. Plant..

[31] Otulak K., Kozieł E., Garbaczewska G. (2016). Ultastructural impact of tobacco rattle virus on tobacco and pepper ovary and anther tissues. J. Phytopatol..

[32] Otulak-Kozieł K., Kozieł E., Lockhart B.E.L., Bujarski J.J. (2020). The Expression of Potato *expansin A3* (*StEXPA3*) and extensin4 (*StEXT4*) genes with distribution of StEXPAs and HRGPS-extensin changes as an effect of cell wall rebuilding in two types of PVY^NTN^–*Solanum tuberosum* Interactions. Viruses.

[33] Otulak-Kozieł K., Kozieł E., Lockhart B.E.L. (2018). Plant cell wall dynamics in compatible and incompatible potato response to infection caused by *Potato virus Y* (PVYNTN). Int. J. Mol. Sci..

[34] Otulak-Kozieł K., Kozieł E., Bujarski J.J. (2018). Spatiotemporal changes in xylan-1/xyloglucan and xyloglucan xyloglucosyl transferase (Xth-Xet5) as a step-in of ultrastructural cell wall remodelling in potato–*Potato virus Y* (PVYntn) hypersensitive and susceptible reaction. Int. J. Mol. Sci..

[35] Kozieł E., Otulak-Kozieł E., Bujarski J.J. (2020). Modifications in tissue and cell ultrastructure as elements of immunity-like reaction in Chenopodium quinoa against Prune dwarf Virus (PDV). Cells.

[36] Clark M.F., Adams A.N. (1977). Characteristics of the microplate method of enzyme-linked immunosorbent assay for the detection of plant viruses. J. Gen. Virol..

[37] Bioreba Company Site. http://www.bioreba.ch/saas/CustomUpload/374O357O340O370O356O369O350O321O360O366O369O356O353O352O350O320O326O/Simple_ELISA_Data_Analysis.pdf.

[38] Otulak-Kozieł K., Kozieł E., Bujarski J.J., Frankowska-Łukawska J., Torres M.A. (2020). Respiratory Burst Oxidase Homologs RBOHD and RBOHF as key modulating components of response in Turnip mosaic virus—*Arabidopsis thaliana* (L.) Heyhn System. Int. J. Mol. Sci..

[39] Liu J., Pang X., Cheng Y., Yin Y., Zhang Q., Su W., Hu B., Guo Q., Ha S., Zhang J. (2018). The *Hsp70* Gene Family in *Solanum tuberosum*: Genome-wide identification, phylogeny, and expression patterns. Sci. Rep..

[40] Luschin-Ebengreuth N., Zechmann B. (2016). Compartment-specific investigations of antioxidants and hydrogen peroxide in leaves of Arabidopsis thaliana during dark-induced senescence. Acta Physiol. Plant..

[41] Wu Y., Eghbali M., Ou J., Lu R., Toro L., Stefani E. (2010). Quantitative determination of spatial protein-protein correlations in fluorescence confocal microscopy. Biophys. J..

[42] Manders E.M., Stap J., Aten J.A. (1992). Dynamics of three-dimensional replication patterns during the S-phase, analyzed by double labeling of DNA and confocal microscopy. J. Cell Sci..

[43] Kozieł E., Otulak K., Lockhart B.E.L., Garbaczewska G. (2017). Subcelullar localization of proteins associated with *Prune dwarf virus* replication. Eur. J. Plant. Pathol..

[44] Kozieł E., Otulak-Kozieł K., Bujarski J.J. (2018). Ultrastructural analysis of *Prune dwarf virus* intercellular transport and pathogenesis. Int. J. Mol. Sci..

[45] Mayhew T.M. (2011). Quantifying immunogold localization on electron microscopic thin sections: A compendium of new approaches for plant cell biologists. J. Exp. Bot..

[46] GraphPad Software Official Website. https://www.graphpad.com/quickcalcs/contingency1.cfm.

[47] Mayhew T.M., Lucocq J.M. (2011). Multiple-labelling immunoEM using different sizes of colloidal gold: Alternative approaches to test for differential distribution and colocalization in subcellular structures. Histochem. Cell Biol..

[48] Hafrén A., Hofius D., Rönnholm G., Sonnewald U., Mäkinen K. (2010). HSP70 and Its cochaperone CPIP promote Potyvirus Infection in *Nicotiana benthamiana* by regulating viral coat protein functions. Plant. Cell.

[49] Lõhmus A., Hafrén A., Mäkinen K. (2017). Coat protein regulation by CK2, CPIP, HSP70, and CHIP is required for Potato Virus A replication and coat protein accumulation. J. Virol..

[50] Valli A.A., Gallo A., Rodamilans B., López-Moya J.J., García A. (2018). The HCPro from the *Potyviridae* family: An enviable multitasking helper component that every virus would like to have. Mol. Plant. Pathol..

[51] De Oliveira L.C., Volpon L., Rahardjo A.K., Osborne M.J., Culjkovic-Kraljacic C., Trahan C., Oeffinger M., Kwok B.H., Borden K.L.B. (2019). Structural studies of the eIF4E-VPg complex reveal a direct competition for capped RNA: Implications for translation. Proc. Natl. Acad. Sci. USA.

[52] Michel V., Julio E., Candresse T., Cotucheau J., Decorps C., Volpatti R., Moury B., Glais L., Dorlhac de Borne F., Decroocq V. (2018). NtTPN1: A RPP8-like R gene required for *Potato virus Y*-induced veinal necrosis in tobacco. Plant. J..

[53] Calil I.P., Fontes E.P.B. (2017). Plant immunity against viruses: Antiviral immune receptors in focus. Ann. Bot..

[54] Yang H., Gou X., He K., Xi D., Du J., Lin H., Li J. (2010). BAK1 and BKK1 in *Arabidopsis thaliana* confer reduced susceptibility to *Turnip crinkle virus*. Eur. J. Plant. Pathol..

[55] Korner C.J., Klauser D., Niehl A., Dominguez-Ferreras A., Chinchilla D., Boller T., Heinlein M., Hann D.R. (2013). The immunity regulator BAK1 contributes to resistance against diverse RNA viruses. Mol. Plant. Microbe Interact..

[56] Niehl A., Wyrsch I., Boller T., Heinlein M. (2016). Double-stranded RNAs induce a pattern-triggered immune signaling pathway in plants. New Phytol..

[57] Niehl A., Heinlein M. (2019). Perception of double-stranded RNA in plant antiviral immunity. Mol. Plant. Phytol..

[58] Valkonen J.P.T. (2015). Elucidation of virus-host interactions to enhance resistance breeding for control of virus diseases in potato. Breed. Sci..

[59] Otulak-Kozieł K., Kozieł E., Valverde R.A. (2019). The Respiratory burst oxidase homolog D (RbohD) cell and tissue distribution in potato–*Potato virus Y* (PVY^NTN^) hypersensitive and susceptible reactions. Int. J. Mol. Sci..

[60] Valkonen J.P.T., Gebhardt C.H., Zimnoch-Guzowska E., Watanabe K.N., Lacomme C., Glais L., Bellstedt D., Dupuis B., Karasev A., Jacquot E. (2017). Resistance to *Potato virus Y* in potato. Potato virus Y: Biodiversity, Pathogenicity, Epidemiology and Management.

[61] Adams M.J., Antoniw J.F., Fauquet C.M. (2005). Molecular criteria for genus and species discrimination within the family Potyviridae. Arch. Virol..

[62] Li X.H., Valdez P., Olvera R.E., Carrington J.C. (1997). Functions of the tobacco etch virus RNA polymerase (NIb): Subcellular transport and protein-protein interaction with VPg/proteinase (NIa). J. Virol..

[63] Mine A., Okuno T. (2012). Composition of plant virus RNA replicase complexes. Curr. Opin. Virol..

[64] Wei T., Huang T.S., McNeil J., Laliberte J.F., Hong J., Nelson R.S., Wang A. (2010). Sequential recruitment of the endoplasmic reticulum and chloroplasts for plant potyvirus replication. J. Virol..

[65] Ferrer-Orta C., Arias A., Escarmis C., Verdaguer N. (2006). A comparison of viral RNA-dependent RNA polymerases. Curr. Opin. Struct. Biol..

[66] Bruenn J.A. (2003). A structural and primary sequence comparison of the viral RNA-dependent RNA polymerases. Nucleic Acids Res..

[67] Li X.H., Carrington J.C. (1995). Complementation of tobacco etch potyvirus mutants by active RNA polymerase expressed in transgenic cells. Proc. Natl. Acad. Sci. USA.

[68] Shatskaya G.S., Drutsa V.L., Koroleva O.N., Osterman I.A., Dmitrieva T.M. (2013). Investigation of activity of recombinant mengovirus RNA-dependent RNA polymerase and its mutants. Biochemistry.

[69] Zheng L., Wayper P.J., Gibbs A.J., Fourment M., Rodoni B.C., Gibbs M.J. (2008). Accumulating variation at conserved sites in potyvirus genomes is driven by species discovery and affects degenerate primer design. PLoS ONE.

[70] Vives-Adrian L., Lujan C., Oliva B., van der Linden L., Selisko B., Coutard B., Canard B., van Kuppeveld F.J., Ferrer-Orta C., Verdaguer N. (2014). The crystal structure of a cardiovirus RNA-dependent RNA polymerase reveals an unusual conformation of the polymerase active site. J. Virol..

[71] Grangeon R., Cotton S., Laliberte J.F. (2010). A model for the biogenesis of turnip mosaic virus replication factories. Commun. Integr. Biol..

[72] Ivanov K.I., Eskelin K., Lõhmus A., Mäkinen K. (2014). Molecular and cellular mechanisms underlying potyvirus infection. J. Gen. Virol..

[73] Wu X., Valli A., García J.A., Zhou X., Cheng X. (2019). The tug-of-war between plants and viruses: Great progress and many remaining questions. Viruses.

[74] Janzac B.F., Fabre M.F., Palloix A., Moury B. (2009). Phenotype and spectrum of action of the *Pvr4* resistance in pepper against potyviruses, and selection for virulent variants. Plant. Pathol..

[75] Janzac B., Montarry J., Palloix A., Navaud O., Moury B. (2010). A point mutation in the polymerase of *Potato virus Y* confers virulence toward the *Pvr4* resistance of pepper and a high competitiveness cost in susceptible cultivar. Mol. Plant. Microbe. Interact..

[76] Kim S.B., Lee H.Y., Choi E.H., Park E., Kim J.H., Moon K.B., Kim H.S., Choi D. (2018). The coiled-coil and leucine-rich repeat domain of the potyvirus resistance protein Pvr4 has a distinct role in signaling and pathogen recognition. Mol. Plant. Microbe Interact..

[77] Fellers J.P., Tremblay D., Handest M.F., Lommel S.A. (2002). The *Potato virus Y* M^S^N^R^ NIb-replicase is the elicitor of a veinal necrosis-hypersensitive response in root knot nematode resistant tobacco. Mol. Plant. Pathol..

[78] Li F., Zhang C., Li Y., Wu G., Hou X., Zhou X., Wang A. (2018). Beclin1 restricts RNA virus infection in plants through suppression and degradation of the viral polymerase. Nat. Commun..

[79] Zhang M., Gong P., Ge L., Chang Z., Cheng X., Zhou X., Wang A., Li F. (2021). Nuclear exportin 1 facilitates turnip mosaic virus infection by exporting the sumoylated viral replicase and by repressing plant immunity. New Phytol..

[80] Otulak K., Garbaczewska G. (2014). The participation of plant cell organelles in compatible and incompatible *Potato virus Y*-tobacco and-potato plant interaction. Acta Physiol. Plant..

[81] Wei T., Wang A. (2008). Biogenesis of cytoplasmic membranous vesicles for plant potyvirus replication occurs at endoplasmic reticulum exit sites in a COPI- and COPII-dependent manner. J. Virol..

[82] Movahed N., Sun J., Vali H., Laliberte J.F., Zheng H. (2019). A host ER fusogen is recruited by turnip mosaic virus for maturation of viral replication vesicles. Plant. Physiol..

[83] Wei T., Zhang C., Hou X., Sanfacon H., Wang A. (2013). The SNARE protein Syp71 is essential for turnip mosaic virus infection by mediating fusion of virus-induced vesicles with chloroplasts. PLoS Pathog..

[84] Martín M.T., García J.A., Cervera M.T., Goldbach R.W., van Lent J.W.M. (1992). Intracellular localization of three non-structural plum pox potyvirus proteins by immunogold labelling. Virus Res..

[85] Li Y., Xiong R., Bernards M., Wang A. (2016). Recruitment of arabidopsis RNA helicase *AtRH9* to the viral replication complex by viral replicase to promote turnip mosaic virus replication. Sci. Rep..

[86] Park S.H., Li F., Renaud J., Shen W., Li Y., Guo L., Cui H., Sumarah M., Wang A. (2017). NbEXPA1, an alpha-expansin, is plasmodesmata-specific and a novel host factor for potyviral infection. Plant. J..

[87] Rodriguez-Peña R., Mounadi K.E., Garcia-Ruiz H. (2021). Changes in subcellular localization of host proteins induced by plant viruses. Viruses.

[88] Li J., Xiang C.Y., Yang J., Chen J.P., Zhang H.M. (2015). Interaction of hsp20 with a viral rdrp changes its sub-cellular localization and distribution pattern in plants. Sci. Rep..

[89] Makarova S., Makhotenko A., Spechenkova N., Love A.J., Kalinina N.O., Taliansky M. (2018). Interactive responses of potato (*Solanum tuberosum* L.) plants to heat stress and infection with *Potato virus Y*. Front. Microbiol..

[90] Hýsková V., Bělonožníková K., Doričová V., Kavan D., Gillarová S., Henke S., Ryšlavá H., Čeřovská N. (2021). Effects of heat treatment on metabolism of tobacco plants infected with *Potato virus Y*. Plant. Biol..

[91] Chen Z., Zhou T., Wu X., Hong Y., Fan Z., Li H. (2008). Influence of cytoplasmic heat shock protein 70 on viral infection of *Nicotiana benthamiana*. Mol. Plant. Pathol..

[92] Boevink P., Oparka K.J. (2005). Virus-host interactions during movement processes. Plant. Physiol..

[93] Kanzaki H., Saitoh H., Ito A., Fujisawa S., Kamoun S., Katou S., Yoshioka H., Terauchi R. (2003). Cytosolic HSP90 and HSP70 are essential components of INF1-mediated hypersensitive response and non-host resistance to Pseudomonas cichorii in Nicotiana benthamiana. Mol. Plant. Pathol..

[94] Gorovits R., Moshe A., Ghanim M., Czosnek H. (2013). Recruitment of the host plant heat shock protein 70 by Tomato yellow leaf curl virus coat protein is required for virus infection. PLoS ONE.

[95] Turner K.A., Sit T.L., Callaway A.S., Allen N.S., Lommel S.A. (2004). Red clover necrotic mosaic virus replication proteins accumulate at the endoplasmic reticulum. Virology.

[96] Wang X., Cao X., Liu M., Zhang R., Zhang X., Gao Z., Zhao X., Xu K., Li D., Zhang Y. (2018). Hsc70-2 is required for *Beet black scorch virus* infection through interaction with replication and capsid proteins. Sci. Rep..

[97] Huang Y.P., Chen I.H., Tsai C.H. (2017). Host factors in the infection cycle of bamboo mosaic virus. Front. Microbiol..

