# Supplementary material for: Modulation of Expression of PVYNTN RNA-Dependent RNA Polymerase (NIb) and Heat Shock Cognate Host Protein HSC70 in Susceptible and Hypersensitive Potato Cultivars
Source: Vaccines (Basel). 2021 Oct 29;9(11):1254. doi: 10.3390/vaccines9111254 (PMC8619674; doi:10.3390/vaccines9111254)
Supplement: Supplementary file 1 [file vaccines-09-01254-s001.zip › vaccines-1410274-supplementary.pdf]

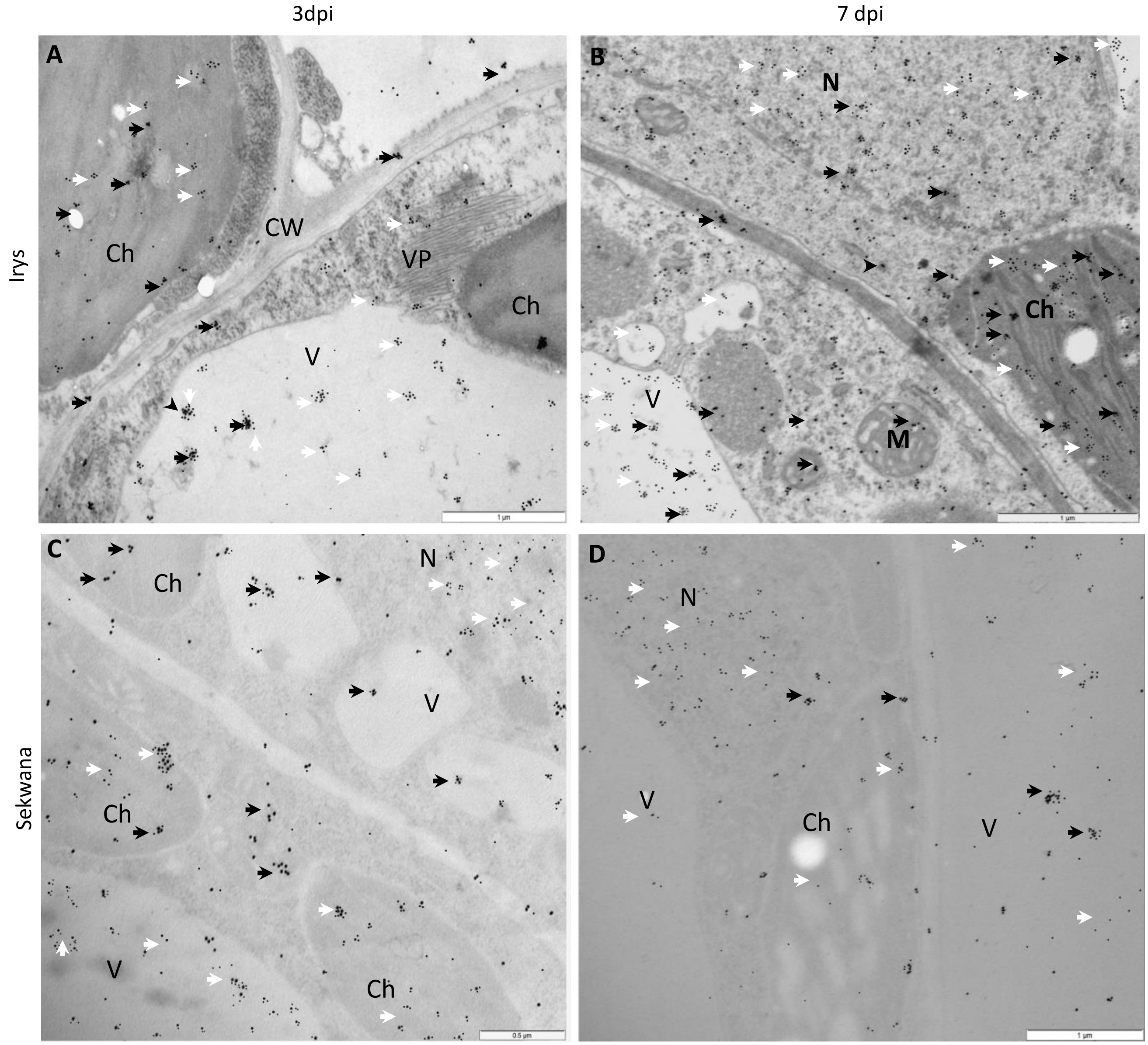

Figure S1. Double immunogold labelling of PVY-NIb (<u>black arrowhead</u>) and HSC70 (<u>white arrowhead</u>) in susceptible (**A-B**) **and resistance** (**C-D**) PVY^{NTN}-potato interactions. (**A**) PVY-NIb with HSC70 deposition in chloroplast (Ch) and vacuole (V) in mesophyll cells. Virus particles (VP) present in cytoplasm. Scale bar 1µm. (**B**) Colocalisation NIb with HSC70 in nucleus (N), inside chloroplast (Ch) and vacuoles (V) in spongy mesophyll cells during susceptible interaction. Scale bar 1µm. (**C**) Colocalisation PVY-NIb with HSC70 in nucleus (N), chloroplast (Ch) and (vacuoles) in palisadę mesophyl cel during hypersensitive response. Scale bar 0.5µm. (**D**) Colocalisation NIb and HSC70 in nucelus (N) and vacuole (V) in phloem parenchyma cell. Scale bar 1µm.

Table S1. Quantification of preferential double-immunogold localization of NIb and Hsc 70 in 2 × 2 contingency table from GraphPad Software. Quantification of double-immunolocalization parameters in: PVY ^{NTN} inoculated cv Irys at 3 dpi (A) and 7 dpi and also PVY NTN inoculated cv Sekwana at 3 dpi (C) and 7 dpi (D). Two-tailed *p* value (*p*) for cell segments analyses was less than 0.0001. OR—odds ratio, NIb$_{g18}$+—presence of 18 nm gold particles associated with presence of NIb epitope, NIb$_{g18}$−—absence of 18 nm gold particles associated with presence of NIb epitope, HSC 70$_{g10}$+—presence of 10 nm gold particles associated with presence of HSC 70 epitope, HSC 70$_{g10}$−—absence of 10 nm gold particles associated with presence of HSC 70 epitope. In table bold and red color value is result of statistical analyses of quantification of preferential double-immunogold localization.

Double-Immunolocalization Parameters

**(A) PVY ^{NTN} inoculated cv Irys 3 dpi:**				
**vacuole**				
Protein	Hsc 70 g10$^+$	Hsc 70 g10$^-$	Row totals	Ratio HSC70$_{g10+}$/HSC70$_{g10-}$
NIb g18$^+$	74	9	83	8.22
NIb g18$^-$	4	12	16	0.33
Column totals	78	21	99	**OR=24.68**
**nucleus**				
Protein	Hsc 70 g10$^+$	Hsc 70 g10$^-$	Row totals	Ratio HSC70$_{g10+}$/HSC70$_{g10-}$
NIb g18$^+$	84	5	89	16,8
NIb g18$^-$	7	13	20	0.54
Column totals	91	18	109	**OR=31.2**
**chloroplast**				
Protein	Hsc 70 g10$^+$	Hsc 70 g10$^-$	Row totals	Ratio HSC70$_{g10+}$/HSC70$_{g10-}$
NIb g18$^+$	30	5	35	6
NIb g18$^-$	2	7	9	0.29
Column totals	32	12	44	**OR=21**
**(B) PVY ^{NTN} inoculated cv Irys 7 dpi**				
**vacuole**				
Protein	Hsc 70 g10$^+$	Hsc 70 g10$^-$	Row totals	Ratio HSC70$_{g10+}$/HSC70$_{g10-}$
NIb g18$^+$	90	3	93	30
NIb g18$^-$	3	5	8	0.6
Column totals	93	8	101	**OR=50**
**nucleus**				
Protein	Hsc 70 g10$^+$	Hsc 70 g10$^-$	Row totals	Ratio HSC70$_{g10+}$/HSC70$_{g10-}$
NIb g18$^+$	109	5	114	21.8
NIb g18$^-$	6	21	27	0.29
Column totals	115	26	141	**OR=76.3**
**chloroplast**				
Protein	Hsc 70 g10$^+$	Hsc 70 g10$^-$	Row totals	Ratio HSC70$_{g10+}$/HSC70$_{g10-}$
NIb g18$^+$	81	5	86	16.2
NIb g18$^-$	3	7	10	0.44
Column totals	84	12	96	**OR=37.8**
**(C) PVY ^{NTN} inoculated cv Sekwana 3 dpi**				
**vacuole**				
Protein	Hsc 70 g10$^+$	Hsc 70 g10$^-$	Row totals	Ratio HSC70$_{g10+}$/HSC70$_{g10-}$
NIb g18$^+$	60	14	74	4.29
NIb g18$^-$	3	9	12	0.33
Column totals	63	23	86	**OR=12.86**
**nucleus**				
Protein	Hsc 70 g10$^+$	Hsc 70 g10$^-$	Row totals	Ratio HSC70$_{g10+}$/HSC70$_{g10-}$
NIb g18$^+$	85	10	95	8.5
NIb g18$^-$	6	13	19	0.46
Column totals	91	23	114	**OR=18.42**
**chloroplast**				
Protein	Hsc 70 g10$^+$	Hsc 70 g10$^-$	Row totals	Ratio HSC70$_{g10+}$/HSC70$_{g10-}$
NIb g18$^+$	40	5	45	8
NIb g18$^-$	3	4	7	0.75
Column totals	43	9	52	**OR=10.67**
**(D) PVY ^{NTN} inoculated cv Sekwana 7 dpi**				
vacuole				
Protein	Hsc 70 g10$^+$	Hsc 70 g10$^-$	Row totals	Ratio HSC70$_{g10+}$/HSC70$_{g10-}$
NIb g18$^+$	36	10	46	3.6
NIb g18$^-$	6	10	16	0.6
Column totals	42	20	62	**OR=6**
nucleus				
Protein	Hsc 70 g10$^+$	Hsc 70 g10$^-$	Row totals	Ratio HSC70$_{g10+}$/HSC70$_{g10-}$
NIb g18$^+$	38	9	47	4.22
NIb g18$^-$	6	11	17	0.55
Column totals	44	20	64	**OR=7.74**

chloroplast				
Protein	Hsc 70 g10$^+$	Hsc 70 g10$^-$	Row totals	Ratio $HSC70_{g10+}/HSC70_{g10}-$
NIb g18$^+$	13	5	18	2.6
NIb g18$^-$	3	5	8	0.6
Column totals	16	10	26	**OR=4.33**
